# Fine-tuned GPT-based foundation models effectively reconstruct bacterial transcriptional regulatory networks from literature

**DOI:** 10.3389/frai.2026.1788196

**Published:** 2026-04-13

**Authors:** José Romero-Vilchis, Maximiliano Barajas-Sánchez, Karyme-Ivette Azpeitia-García, Ali-Berenice Posada-Reyes, Julio Collado-Vides, Carlos-Francisco Méndez-Cruz

**Affiliations:** 1 Programa de Genómica Computacional, Centro de Ciencias Genómicas, Universidad Nacional Autónoma de México (UNAM), Cuernavaca, Morelos, Mexico; 2 Facultad de Ciencias, Universidad Autónoma del Estado de México, Toluca, Mexico; 3 Department of Applied Mathematics and Systems, Universidad Autónoma Metropolitana, Cuajimalpa Campus, Mexico City, Mexico; 4 Faculty of Sciences, National Autonomous University of Mexico, Mexico City, Mexico; 5 Universidad Nacional Autónoma de México, Ciudad de México, Mexico; 6 Centre for Genomic Regulation (CRG), Universitat Pompeu Fabra (UPF), Barcelona, Spain; 7 Department of Biomedical Engineering, Boston University, Boston, MA, United States

**Keywords:** foundation model, GPT, LLaMA, NLP, regulatory network, *Salmonella*, supervised fine-tuning, transcriptional regulation

## Abstract

**Introduction:**

Life has the property to produce from a single genome, the collection of DNA molecules, different cell types, as well as mechanisms for bacteria to adapt to environmental changes. Although regulation can happen at different levels, regulation of transcription initiation, the start of copying DNA into RNA, is the most studied level in bacteria. The collection of regulators and their regulated elements defines transcriptional regulatory networks (TRNs), whose study has driven relevant areas, such as antimicrobial resistance. Their analyses and understanding depend on some few highly manually curated databases. The traditional way to reconstruct these networks is by manual curation of the literature, which is accurate, but also demanding and time-consuming. These limitations have resulted in the shortage and incompleteness of bacterial TRNs.

**Methods:**

Here, we present a novel ensemble model approach using two GPT-based foundation models (LLaMA-3 and GPT-4o mini) to effectively reconstruct TRNs from the literature. We applied a supervised fine-tuning strategy with sentences from *Escherichia coli* literature to train models to predict the type of regulatory effect between a transcription factor and a regulated element (gene/operon). To evaluate the performance of reconstructing a curated TRN, we used 264 full-text articles of *Salmonella* Typhimurium, a pathogen of clinical interest.

**Results:**

With the test data, both models obtained significant performance (F1-Score > 0.87, Matthews correlation coefficient > 0.82). For the curated TRN reconstruction, the ensemble approach using the agreement of models correctly reconstructed 80% of the TRN (Recall: 0.80, F1-score: 0.64). We applied the approach to reconstruct a large *Salmonella* TRN using the literature available at the time on transcriptional regulation of this bacterium (2,278 articles). This network was described with network metrics, over-representation analyses, and compared to existing biological knowledge.

**Discussion:**

Our approach overtook the performance of prior works predicting the effect of the interaction. The analysis of the TRN of the 2,278 articles showed the effectiveness of our approach to reconstruct TRNs of diverse bacteria, as the network aligns with biological knowledge. Thus, our work may support the study of bacteria of biological and clinical interest, especially those without a reconstructed TRN.

## 1 Introduction

The bacterial genome is controlled by different regulatory mechanisms that enhance or inhibit gene expression, the best studied level of regulation is *transcriptional regulation*. Through gene expression, bacteria can respond to internal or environmental signals (Fang et al., 2017). This response occurs primarily through the action of proteins called *transcription factors* (TFs), which activate or repress the expression of target genes or a group of genes (operon) to induce response mechanisms (Balleza et al., 2008). For example, in the *Escherichia coli* bacterium (*E. coli*), high oxygen concentrations act as a signal to initiate aerobic respiration by activating or repressing certain genes (Freyre-Gonzalez and Treviño-Quintanilla, 2010). These regulatory mechanisms can be represented by *transcriptional regulatory networks* (TRNs), which describe the general organization of transcriptional regulation in an organism and display hierarchical and modular characteristics due to their network representation (Balleza et al., 2008). The set of transcriptional regulatory interactions TF-gene/operon constitutes a bacterial TRN (Madan Babu and Teichmann, 2003).

The study of TRNs is crucial to understand the different relevant adaptation mechanisms that bacteria have to environmental challenges. For example, the mechanisms detailed in an *E. coli* TRN have been described to play an important role in the survival of this bacterium to colonize the large intestine within its hosts (Yamamoto, 2014). From an evolutionary perspective, Dalldorf et al. (2024) discovered in laboratory experiments that *E. coli* can face short-term environmental challenges through genetic regulation of the TRN despite mutations applied to TFs. A relevant benefit of the study of bacterial TRNs is the identification of regulatory mutations associated with drug resistance, such as those identified in the *marR* gene in clinical isolates of *E. coli* resistant to ciprofloxacin (Praski Alzrigat et al., 2017). The *marR* gene encodes the MarR protein, which is a TF repressing the transcription of various genes responsible for the drug resistance mechanism.

Other studies have described the gene regulation in clinically important bacteria responsible for infections in humans or animals, and their association with important implications for disease severity. An example of this is reported by Shah (2013) for *Salmonella enterica* serovar Enteritidis in poultry strains. *Salmonella* is a bacterium that causes food-borne bacterial gastroenteritis worldwide. In that work, author studied 252 differentially expressed genes in low-pathogenic strains compared to their expression in highly pathogenic strains and found that low-pathogenic genes display a unique transcriptional profile characterized by reduced expression of genes involved in virulence (fimbrial, motility, and stress-associated genes). Characterizing these types of regulatory mechanism would help researchers identify strains with relevant virulence, which can subsequently be attenuated and become vaccine candidates against the bacteria (Shah, 2013). Thus, making bacterial TRNs accessible to the scientific community has important implications for increasing biological and clinical knowledge, which may eventually have social impact. Furthermore, the study of TRNs may have applications in the pharmaceutical field, with the potential development of vaccines or the identification of genes with virulence functions that can subsequently be targeted by drugs. It is important to mention that a different approach to study drug response using gene expression is by creating networks from gene expression profiles leveraging public omics data. For example, Sefer (2025) proposed a deep learning approach for drug response prediction using a graph attention network (DRGAT).

Nowadays, some biological databases are available on digital platforms to freely access bacterial TRNs, such as RegulonDB (Salgado et al., 2024), which contains the TRN of *E. coli* K-12, a model organism for microbiology studies; RegulomePA, which reports the TRN of *Pseudomona aeruginosa* PAO1 (Galán-Vásquez et al., 2020); and *SalmoNet2*, which reports TRNs of several *Salmonella* strains (Métris et al., 2017); among other databases. Their objective is to integrate knowledge of the different regulatory mechanisms of bacteria into increasingly complete networks. However, despite these and other efforts, the TRNs published to date are still incomplete or non-existent for many bacteria (Escorcia-Rodríguez et al., 2020). This is due to the lack of the experimentally identified TRNs in many bacteria, or, for those already identified, the traditional procedure to integrate the TRN has been the manual extraction of regulatory interactions from collections of articles (the so-called *biomedical curation of the literature*), that in some cases accumulate years of curation work (International Society for Biocuration, 2018).

The biomedical curation of the literature consists of the identification of relevant publications and the selective extraction of knowledge pieces for their integration and display in databases (Davies, 2025). This enables to obtain information in a very precise way, but has the disadvantage of being demanding and time-consuming (Howe et al., 2008). In addition, it faces the challenge of accelerated scientific production (Bornmann et al., 2021). Given these limitations and challenges, the adoption of Natural Language Processing (NLP) and Artificial Intelligence (AI) approaches to facilitate and promote the work of extracting and integrating biomedical knowledge from the literature has long been essential (Ananiadou et al., 2014; Huang and Lu, 2015; Zhao et al., 2021).

There are two common NLP tasks to extract knowledge from the biomedical literature: Named Entity Recognition (NER) and Relation Extraction (RE) (Zhao et al., 2021). The first task involves identifying, annotating and normalizing names of biological or biomedical entities in documents, such as proteins, genes, drugs, or diseases. NER is applied in extracting key biological concepts, which helps to build ontologies and knowledge bases. The RE task detects whether entities have a relationship, such as protein-protein interaction, gene-disease association, genotype-phenotype relation, or drug-disease interaction (Zhao et al., 2021; Zhang et al., 2024).

Here, we present an ensemble model approach that surpasses existing approaches to effectively reconstruct TRNs from the literature. A main novelty of our work is the reconstruction of TRNs using foundation models based on Generative pre-trained transformers (GPTs) (Radford et al., 2018), which have not been studied for this specific task until now. Leveraging the knowledge learned in the pre-training step of two GPT-based foundation models (LLaMA-3 and GPT-4o mini), we applied a supervised fine-tuning strategy with 1562 sentences from the literature of *E. coli* to obtain two fine-tuned models able to predict with relevant scores the type of regulatory effect between a TF and a regulated element (gene/operon). The prediction of the effect is another novelty of our work, as the prevailing approach is to predict only if the interaction is true or false (Gill et al., 2024).

In addition, we used 264 full-text articles of *Salmonella enterica* serovar Typhimurium (*Salmonella*), a pathogen of clinical interest as it is the leading cause of food-borne illness (Fábrega and Vila, 2013), to evaluate the reconstruction of a TRN curated from the same articles. An ensemble approach of the two fine-tuned models showed the best performance for reconstructing the TRN. Thus, we used this approach for the reconstruction of a TRN from the literature available at the time on transcriptional regulation of this bacterium (2,278 articles). This network was described using network metrics, over-representation analyses, and compared to well-known biological knowledge to evaluate its consistency. Both TRNs are openly published on a Web platform. Our work shows the potential to integrate regulatory interactions dispersed in the literature to reconstruct a biological network that may be useful to support curation work, validate *in silico* predictions and reveal relevant patterns. Eventually, our work may improve the knowledge of bacteria of biological and clinical interest.

## 2 Materials and methods

### 2.1 Data sets for the study

#### 2.1.1 Data set for fine-tuning

For fine-tuning the GPT-based models and comparing our results with previous work, we leveraged the data set of sentences from 119 journal articles of the *E. coli* literature presented in the work of Varela-Vega et al. (2024). The data set comprises 1,562 sentences in the appropriate format for fine-tuning the GPT-based models. Authors pre-processed sentences with the following procedure:

1. A NER process of TFs and regulated elements was performed for all sentences using lists of names of both entities (dictionaries).
2. The sentences were repeated for all combinations of one TF and one regulated element.
3. All sentences were manually classified according to the regulatory effect of TF on the regulated element as “activator,” “repressor,” “regulator,” or “no_relation.”

The categories of regulatory effects refer to the categorization of the RegulonDB database, the main open access TRN of *E. coli* (Salgado et al., 2024). The “regulator” category is employed when the regulatory effect exists, but the type of effect is not clearly reported, for example, in the following sentence.

- “acrZ is regulated by MarA, Rob, and SoxS.” (Hobbs et al., 2012)

The “activator,” “repressor,” and “regulator” categories were assigned only if the regulatory interaction is explicitly expressed for the pair of mentions of the TF and the regulated element; otherwise, “no_relation” was assigned. For example, in row 1 of Table 1 the mentions of the regulated element and the TF (in boldface) explicitly express that the *melR* gene is repressed by the MelR TF, whereas in row 2 the pair of mentions do not explicitly express the interaction.

**Table 1:** Example of sentences for fine-tuning with manually assigned categories.

#	Category	Sentence
1	“Repressor”	We show that the ***melR*** promoter is repressed by **MelR** and that this autoregulation requires MelR binding to Site R.
2	“No_relation”	We show that the ***melR*** promoter is repressed by MelR and that this autoregulation requires **MelR** binding to Site R.

Entity mentions of TFs and genes are in boldface. Sentence recover from (Wade et al. 2000).

To fine-tune the GPT-based models for learning contextual patterns associated with the interaction instead of learning patterns associated with the specific name of entities, entity mentions were *anonymized* using the @TF$ and @Regulated$ pre-defined tags. Using this procedure, we expect that the model may perform accurately in a new data set with different entity names, for example, in the literature of a different bacterium. The following example shows this procedure, which is commonly used to create fine-tuning data sets for RE (Gill et al., 2024; Zhang et al., 2024).

- **Original sentence:** “We show that the ***melR*** promoter is repressed by **MelR** and that this autoregulation requires MelR binding to Site R.” (Wade et al., 2000)
- **Tagged sentence:** “We show that the **@Regulated$** promoter is repressed by **@TF$** and that this autoregulation requires MelR binding to Site R.”

To compare our approach with previous work, we used the same data sets of Varela-Vega et al. (2024). The authors randomly selected 20% of the data for testing (test data set) and randomly split the remaining 80% into 80% for parameter optimization (train data set) and 20% to find the best hyperparameters (dev data set). A 5-fold cross-validation strategy was utilized to measure the consistency of the models. The three data sets kept the same sentence distribution by category as the complete data set (Table 2).

**Table 2:** Distribution of sentences with anonymized entities in data sets for fine-tuning.

Category	Train+Dev	Test	Total
Activator	477	116	593
No_relation	390	103	493
Repressor	214	55	269
Regulator	168	39	207
	1,249	313	1,562

#### 2.1.2 Data set to evaluate TRN reconstruction

As our approach aims to reconstruct TRNs of several bacteria using full-text articles, we evaluated the best fine-tuned models of GPT-4o mini and LLaMA-3, and the ensemble model approach for the reconstruction of a *Salmonella* TRN using the 264 curated articles used in a prior work (Varela-Vega et al., 2024). The manual curation work of the articles extracted 909 unique interactions (TF-regulated element-effect), which were compared with the interactions predicted by GPT-4o mini, LLaMA-3, and the ensemble model approach for evaluation.

Varela-Vega et al. (2024) performed the following procedure to obtain the collection of articles. The 264 PDF files were converted to raw text using an in-house extractor. The sentence split and tokenization of text articles were performed using the Stanford CoreNLP tool v3.9.1 (Manning et al., 2014). Using TF and regulated element dictionaries, both entities were recognized in sentences. Then, for each combination of one TF and one regulated element, the sentence was duplicated and the pair of entities was anonymized using the pre-defined tags. A total of 14349 tagged sentences were available for the best GPT-4o mini and LLaMA-3 models to predict the regulatory effect. The evaluation of the TRN reconstruction was performed by comparing the predicted regulatory interactions (TF-regulated element-effect) with the regulatory interactions manually extracted from the same 264 articles.

### 2.2 GPT-based foundation models

A Foundation Model is a model that is pre-trained with a great amount of data that can be fine-tuned to solve a downstream task with limited data (Bommasani et al., 2021). A common architecture of artificial neural networks used to develop foundation models is the transformer (Vaswani et al., 2017). When the foundation model is pre-trained with text data (literature), this is called a Large Language Model (LLM) (Kalyan et al., 2022). Research to extend the application of pre-trained language models is a growing area not only for genomics but also in other areas such as finance where significant progress has been shown (Ergun and Sefer, 2025). The pre-training is a self-supervised learning step, where the parameters of all transformer layers are optimized to find a contextual representation of all input tokens, which is learned by a multi-headed self-attention approach (Vaswani et al., 2017). A token is a piece of input text, such as a word, a word segment, or a symbol. The contextual representation encodes relational patterns among distance tokens, which has been successfully leveraged to address several NLP tasks. The fine-tuning is a supervised learning step, where limited annotated data is used for the pre-trained transformer to adjust some parameters to solve a task (Radford et al., 2018). The fine-tuning step not only has the benefit of using limited data for solving the task, but also takes less learning time (less epochs) than pre-training and a general-purpose foundation model can be fine-tuned for a domain-specific task. Here, we fine-tuned two general-purpose foundational models for the domain-specific task of reconstructing a TRN from biomedical literature: the open model LLaMA-3 (Touvron et al., 2023) and the close model GPT-4o mini (Radford et al., 2019).

#### 2.2.1 GPT-4o mini

The GPT-4o mini is a multi-modal model developed by OpenAI that represents a highly efficient and compact version of its GPT-4o line (OpenAI, 2024b). This model stands out for its balance between performance and cost, offering low-latency responses. It features a context window of up to 128,000 tokens and can generate up to 16,000 output tokens per request, facilitating applications that require extensive contextual input. In benchmark evaluations, GPT-4o mini has outperformed other small models such as Gemini Flash and Claude Haiku in textual reasoning tasks (82.0% for Massive Multitask Language Understanding), mathematical reasoning (87.0% for Multi-Step Grade School Math) and programming (87.2% for HumanEval). It also utilizes an improved tokenizer shared with GPT-4o, which enhances cost efficiency in the handling of multilingual text. Its low inference cost ($ 0.15 per million input tokens and $ 0.60 per million output tokens), together with the fine-tuning pricing ($0.30 per million input tokens, $ 1.20 for output, and $ 3.00 per million tokens during training), make it a highly viable tool for large-scale and research-oriented applications (Zhang et al., 2024; Phan et al., 2024). Fine-tuning GPT-4o mini not only reduces inference latency and prompt length, but also enables training on more examples than can fit in a single prompt, which in turn improves result quality and efficiency (OpenAI, 2024a). It is particularly effective in scenarios where it is advisable to use examples to *show* the model how to behave when learning new tasks, setting a desired tone or adhering to user-defined constraints than *telling* the model how to behave using prompts (OpenAI, 2024a).

#### 2.2.2 LLaMA-3 8B and 4B (4-bit LoRA)

The LLaMA-3 8B-Instruct and its lighter LLaMA-3 4B-Instruct companion checkpoints released by Unsloth are decoder-only transformers that natively conform to Meta's chat-ML schema (Unsloth AI, 2025). The 8B variant comprises 32 layers and 8.03 billion parameters, whereas the 4B model contains roughly half that capacity (24 layers, 4.29 billion parameters). For both backbones, all frozen weights are loaded in block-quantized 4-bit INT format using *bitsandbytes*, achieving a ~75% reduction in GPU memory while preserving baseline perplexity (Dettmers et al., 2022). Task-specific adaptability is injected through rank-16 Low-Rank Adaptation (LoRA) applied uniformly to every attention and MLP projection matrix, while the original parameters remain frozen. The resulting artifact—quantized backbone plus LoRA deltas—occupies only 7MB for the 8B model and 4MB for the 4B model, delivering sub-20ms and sub-12ms first-token latency. Optimization employs 8-bit AdamW with a learning rate peak of $\eta_{\max}=2\times 10^{-4}$, linear decay (five warm-up steps), and an effective batch size of eight sequences (2 per device × 4 gradient-accumulation steps). Unless otherwise noted, all hyper-parameters and data pipelines are identical for the 8B and 4B runs. For inference, text generation with LLaMA-3 was performed using the default decoding configuration provided by the Unsloth framework, with the temperature parameter set to 1.0. A temperature of 1.0 corresponds to the neutral setting in probabilistic decoding, preserving the original token probability distribution of the model without additional sharpening or flattening. Unsloth recommends this value as the default configuration to maintain a balance between output diversity and stability during generation, particularly for instruction-tuned models (Unsloth AI, 2024).

### 2.3 Supervised fine-tuning strategy

**Figure 1:**
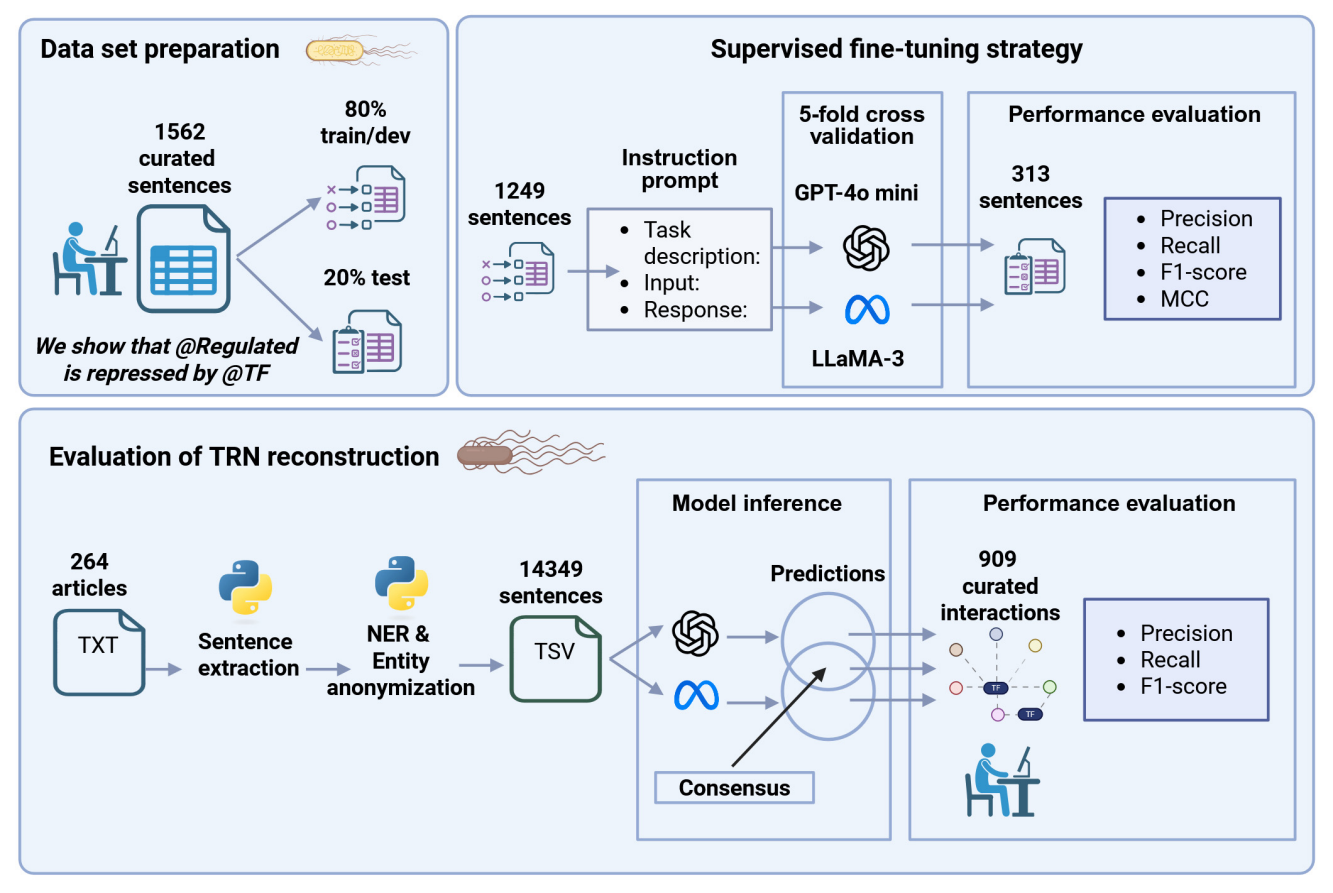
General graphic description of model development including fine-tuning model and performance evaluation. Created in BioRender. Méndez, C. (2026) https://BioRender.com/uac6fdl.

A general graphic description of model development including fine-tuning and performance evaluation is shown in Figure 1.

To ensure comparability, we use the same base instruction prompt to fine-tune both models (Table 3). The *task description* section includes specific instructions, response categories with short descriptions, response constraints to ensure that the model responds with only one category, and the indication of using the “no_relation” category if the model has no answer. As we are using generative models, these aspects are important to limit the *creativity* of the model and force it to give specific answers. The *input* section provides the model with evidence, i.e., sentences of the train data set and the instruction to classify the interaction with a single category. Finally, the *response* section gives the correct category to the model. This base instruction was formatted according to each model format recommendation.

**Table 3:** Base instruction prompt utilized for fine-tuning the GPT-4o mini and LLaMA-3 models.

Instruction prompt example
Task description:
You are an expert in bacterial transcriptional regulation. Analyze the given sentence containing a transcription factor (@TF$) and a regulated element (@Regulated$), and determine the regulatory interaction between the transcription factor and the regulated element. Choose one label: activator (The transcription factor @TF$ promotes the expression of the regulated element @Regulated$), repressor (The transcription factor @TF$ inhibits the expression of the regulated element @Regulated$), regulator (The transcription factor @TF$ regulates the expression of the regulated element @Regulated$, but the regulatory effect is unclear), or no_relation (There is no regulatory interaction between the transcription factor @TF$ and the regulated element @Regulated$).
Base your response only on the provided evidence. Respond with exactly one word from the list: activator, repressor, regulator, or no_relation. If no regulatory effect is clearly indicated, choose no_relation.
Input:
EVIDENCE: *We show that the @Regulated$ promoter is repressed by @TF$*
Based on the provided evidence, determine the regulatory interaction between the transcription factor @TF$ and the regulated element @Regulated$. Respond with exactly one word: activator, repressor, regulator, or no_relation.
Response:
repressor

We used a 5-fold cross-validation strategy to evaluate the consistency of the fine-tuning of the models. Specific strategies for supervised fine-tuning were implemented for the GPT-4o mini and LLaMA-3 following the best practices for each. The fine-tuning of the GPT-4o mini model was carried out using OpenAI API endpoints and the GPT-4o mini-2024-07-18 model snapshot. The data sets were formatted in JSON Lines format (JSONL) according to the OpenAI Chat Completions format. The OpenAI *system role* corresponds to the *Task description* section of the base instruction prompt (Table 3), the OpenAI *user message* to the EVIDENCE of the *Input* section, and the OpenAI *assistant message* to the *Response* section. The temperature parameter was manually set to 0 to ensure a deterministic result in the evaluation of the model. The hyper-parameters were selected automatically by the OpenAI platform when using the OpenAI API, which aims to balance convergence, stability, and resource constraints. The platform consistently used 3 epochs and a small batch size of 1 during cross-validation. For the final fine-tuning run to obtain the best model, we manually set the batch size to 1 to prioritize stability and reproducibility (matching the regime that worked consistently during cross-validation) and to reduce sensitivity to long examples or memory-related variability. This choice allowed us to keep the training configuration unchanged while adopting a more conservative hyperparameter setup for the cross-validation and the final model creation stages.

When it comes to applying fine-tuning techniques to open-source models, such as LLaMA-3, we came across a wide variety of pre-existing frameworks. In the end, given the limited availability of specialized hardware that we faced, we opted for using *Unsloth* since it distributes open-source models with 4-bit quantization already integrated and Low-Rank Adaptation techniques allowing for an efficient usage of our resources. The instruction prompt for each instance was serialized through the Llama-3.1 chat template, producing contexts of up to 2048 tokens. The fine-tuning process was carried out using Unsloth's implementation of the SFTTrainer. Regarding hyper-parameters, we selected a learning rate of 2e-4, a batch size of 2, and gradient accumulation over 4 steps. We used an early stopping policy that terminates training when the model loss does not improve over two consecutive epochs by a margin of at least 2e-5. These choices align with Unsloth's recommended practices for efficient fine-tuning of LLMs. In addition, the model was fine-tuned with mixed precision (fp16 or bf16) to further optimize memory consumption on the available hardware.

For inference with LLaMA-3, we adopted the default decoding parameters recommended by the Unsloth framework. Specifically, generation was performed with a temperature of 1.0, which corresponds to the neutral decoding setting and preserves the original token probability distribution without artificial sharpening or flattening. This choice is recommended by Unsloth as a balanced default that maintains output diversity while ensuring stable and coherent generations, particularly for instruction-tuned models (Unsloth AI, 2024). Top-*p* (nucleus) sampling was set to its default value (*top_p* = 1.0), effectively disabling nucleus truncation and allowing the model to sample from the full probability distribution. This configuration avoids introducing additional bias during inference and is commonly used as a baseline setting in Transformer-based text generation frameworks (Wolf et al., 2020). Similarly, top-*k* sampling was left unconstrained (*top_k* = 0), meaning that no restriction was imposed on the number of candidate tokens considered at each decoding step. Leaving *top_k* disabled is consistent with default decoding behavior and ensures that the model's learned probability distribution is not artificially limited (Wolf et al., 2020). Unless otherwise specified, sampling-based decoding was enabled (*do_sample* = True), which is the default configuration in Unsloth for generative inference. This setting allows probabilistic decoding rather than greedy selection, and is recommended when using temperature-based sampling to preserve linguistic variability and robustness in generated outputs (Wolf et al., 2020; Unsloth AI, 2024).

### 2.4 Ensemble model approach for TRN reconstruction

After fine-tuned models classified the regulatory effect of sentences with anonymized entities, reconstruction of a TRN starts by discarding the sentences classified in the “no_relation” category. Then, we obtained unique regulatory interactions, i.e., a unique combination of TF, regulated element and category (TF-regulated element-effect). When the same TF-regulatory element appeared with different regulatory effects across multiple sentences, all interactions were kept because we have observed that some sentences express an interaction with the “regulatory” effect, whereas others express the same interaction with the specific effect: “activator” or “repressor.” The same decision was made in the case of contradictory effects (“activator” and “repressor”), because one effect could be observed in certain experimental conditions, whereas the other could be observed in a different condition. See the following examples of the interaction between ArcA and *ompD*.

- *Transcript levels of* ***ompD*** *were measured since its expression is* ***regulated*** *by* ***ArcA*** *under ROS conditions*. True category: regulator.
- *We recently demonstrated that* ***ArcA negatively regulates*** *the expression of S. Typhimurium* ***ompD*** *after H2O2 exposure by direct interaction with its promoter region*. True category: repressor.

With the aim of exploring strategies to improve the performance of TRN reconstruction, we evaluated the agreement of the fine-tuned models (*ensemble model approach*). We employed a voting-like strategy, which consisted in considering only the interactions predicted by both models as the final set of predicted interactions. In other words, the final interactions predicted by the ensemble model approach were the consensus of both models; interactions predicted only by one model were discarded.

### 2.5 Performance evaluation

Fine-tuning performance evaluation was achieved by calculating standard metrics for a multiclass classification task: Precision, Recall, and F1-Score Macro (Christopher et al., 2008). These metrics were calculated using the scikit-learn library (Pedregosa et al., 2011). The Precision score is the fraction of examples predicted correctly by the model (Equation 1):


$$\begin{array}{c} \frac{TP}{TP+FP}. \end{array}$$ (1)

The Recall score is the fraction of correctly predicted examples of the total examples (Equation 2), and the F1-Score is the harmonic mean of Precision and Recall (Equation 3). We used the Macro version of the F1-Score, which is calculated first for each category and then averaged, giving the same importance to all categories. The three metrics range from 0 to 1, with 1 being the best value (Christopher et al., 2008):


$$\begin{array}{c} \frac{TP}{TP+FN}, \end{array}$$ (2)

$$\begin{array}{c} 2\frac{Precision\times Recall}{Precision+Recall}. \end{array}$$ (3)

The best models of the GPT-4o mini and LLaMA-3 were determined by comparing the mean F1-score of the cross-validation strategy. Then, the final model of both was obtained by a last run of fine-tuning using the total sentences of the train and the dev data sets together. Finally, we assessed the performance of the final models using the test data set. In the test evaluation, we included the Matthews correlation coefficient (MCC) (Chicco et al., 2021). This metric shows the correlation of the true categories with the predicted categories, ranging from –1 to 1, with 1 being the best value (Equation 4). This coefficient is suggested for problems with imbalanced categories, because it is higher if the model correctly predicts the majority of categories (Chicco et al., 2021):


$$\begin{array}{c} MCC=\frac{Cov\left(c,l\right)}{\sigma_{c}\sigma_{l}} \end{array}$$

$$\begin{array}{cc} = & \frac{\left(TP\times TN\right)-\left(FP\times FN\right)}{\sqrt{\left(TP+FP\right)\times \left(TP+FN\right)\times \left(TN+FP\right)\times \left(TN+FN\right)}}, \end{array}$$ (4)

where *Cov*(*c, l*) is the covariance of the true categories *c* and the predicted categories *l* and σ_*c*_ and σ_*l*_ are the standard deviations, respectively.

The best model of each foundation model and the ensemble model approach were evaluated to reconstruct the curated *Salmonella* TRN with 909 interactions manually curated from 264 full-text articles (see Section 2.1.2). For evaluation, the final interactions predicted by the fine-tuned models and the ensemble model approach were searched in the 909 curated interactions to obtain the F1-Score, Precision, and Recall metrics. For calculating these metrics, the true positives (TP) were the predicted interactions that were present in the curated interactions, the false positives (FP) were the predicted interactions that were not present in the curated interactions, and the false negatives (FN) were the interactions from the curated interactions that were not predicted by the model.

Beyond reporting these metrics, we evaluated whether differences in interaction prediction between models and the ensemble approach were statistically significant using McNemar's test (McNemar, 1947). McNemar's test is a non-parametric method for paired nominal outcomes, designed to compare two classifiers in the same instances when the result is binary (correct vs. incorrect). For applying the test, we re-formulated the *Salmonella* TRN reconstruction as a binary decision problem at the level of unique regulatory interaction. Each unique TF-regulated element interaction in the curated *Salmonella* TRN was treated as one true instance. For each model, an instance was labeled as *correct* if the model predicted the interaction, and *incorrect* otherwise. This yields paired outcomes for two models *A* and *B* on the same set of true interactions *n*. For each pairwise comparison (*A* vs. *B*), we constructed a 2 × 2 table:


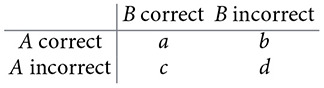


where *b* is the number of instances where *A* is correct and *B* is incorrect, and *c* is the number of instances where *A* is incorrect and *B* is correct. McNemar's test focuses exclusively on *discordant* counts (*b, c*), because *a* and *d* provide no information about differences between models (they agree). McNemar's null hypothesis is as follows:


$$H_{0}:\mathrm{Pr}\left(A\;\text{correct},B\;\text{incorrect}\right)=\mathrm{Pr}\left(A\;\text{incorrect},B\;\text{correct}\right),$$

equivalently *b* and *c* are exchangeable. Under *H*_0_, conditional on the total number of discordant pairs *n*_*d*_ = *b*+*c*, the random variable *b* follows a binomial distribution:


$$b\sim \text{Binomial}\left(n_{d},0.5\right).$$

Thus, the exact two-sided McNemar *p*-value is computed from the binomial tail probabilities. For moderate or large *n*_*d*_, McNemar's test can be approximated by a chi-square distribution with one degree of freedom and continuity correction. All tests were two-sided with α = 0.05.

### 2.6 NER using BERN2

An essential step in the automatic reconstruction of TRNs is the NER of TFs and regulated elements within sentences. In our current approach, we use dictionaries to perform the NER task. To explore if a machine learning-based NER tool can achieve the same annotation of our dictionary-based approach, we obtained the performance of using BERN2 to recognize TFs and genes/operons. BERN2 (Biomedical Entity Recognition and Normalization) is a tool capable of identifying mentions of genes, proteins, diseases, chemical compounds, genetic variants, mutations, and other biologically relevant concepts within scientific texts using deep learning (Sung et al., 2022). We selected BERN2 because it integrates multiple specialized NER models, such as BioBERT, tmVar, and GNormPlus, and combines their outputs through an ensemble approach to improve both Precision and Recall. BERN2 processes biomedical text and returns recognized entities along with their normalized identifiers, linking them to standard databases such as UniProt, MeSH, or Entrez. In this study, we deployed a local installation of BERN2, following the official instructions available in its GitHub repository. This local deployment enabled efficient processing of large textual data sets while eliminating dependencies on external services.

We observed that BERN2 only recognizes genes, i.e., it does not differentiate TFs from genes. Recognizing only genes or proteins is the general approach of all available biomedical NER tools. In addition, we observed that BERN2 recognizes gene mentions including additional lexical items that are not part of the gene/protein name, such as *wild-type ArgP* or *ArgP - binding site*, and recognizes some mentions that are irrelevant for our approach, such as *RNA polymerase*. Therefore, we developed a dedicated algorithm to differentiate TFs from genes and customize mention recognition. The algorithm consists of four stages (BERN2-based NER, tagging sentences, improving entity mentions, and rule-based classification) described in the Supplementary Methods. We refined this algorithm by improving the performance in several iterations using the train data set. To evaluate the final performance, we used the test data set and report metrics. For this evaluation, we used the standard BIO representation for NER tasks. This representation refers to tagging the entity mentions at the beginning with a *B-tag*, at the middle with an *I-tag*, and for non-entities with the *O* tag, for example, the phrase *melR promoter is repressed by MelR* is tagged as *melR/B-GENE promoter/O is/O repressed/O by/O MelR/TF*.

## 3 Results

### 3.1 Fine-tuning performance

To determine the best fine-tuned model of both GPT-based foundation models, we considered the mean of the F1-score Macro calculated with the five runs of cross validation. According to the mean and standard deviation, both models performed similarly (Table 4). The standard deviation of F1-Score showed that LLaMA-3 may be slightly more consistent than GPT-4o mini. Precision, Recall, and MCC scores with standard deviations and confidence intervals for the best models are shown in Supplementary Table S1. As expected, only a few epochs were required to have a good model, which is an advantage of the fine-tuning strategy. Comparing the scores of LLaMA models, a larger model seems more effective for our task, since LLaMA 8B outperforms its 3B version.

**Table 4:** Fine-tuning F1-score Macro and hyperparameters obtained with 5-fold cross validation.

Model	F1-score	Epochs	Batch size	Learning rate
GPT-4o mini	0.886 ± 0.0189	3	1	auto
LLaMA-3 8B - Instruct bnb 4-bit	0.886 ± 0.0169	5	2	2e-4
LLaMA-3 3B - Instruct bnb 4-bit	0.873 ± 0.0149	5	2	2e-4

Auto refers to the original pre-training learning rate used by OpenAI.

Evaluation with the test data set showed that LLaMA 8B surpassed GPT-4o mini in all metrics (Table 5). It should be noted that the score obtained by LLaMA 8B in the dev data set increased in the test data set, showing a lack of over-fitting. According to the MCC, both models obtained a significant correlation between the true categories and the predicted categories. Notice that Both models surpassed metrics reported in previous work for the same task using BERT-based models (Varela-Vega et al., 2024). The fact that the GPT-based models performed better than the BERT-based models is most likely due to the fact that the former have been improved by their companies over time.

**Table 5:** Evaluation metrics obtained with the test data set.

Model	F1-score	Precision	Recall	MCC
LLaMA 8B - Instruct bnb 4-bit	0.892	0.886	0.898	0.852
GPT-4o mini	0.873	0.869	0.886	0.826
BERT-based model (Varela-Vega et al., 2024)	0.868	0.860	0.879	0.816

**Figure 2:**
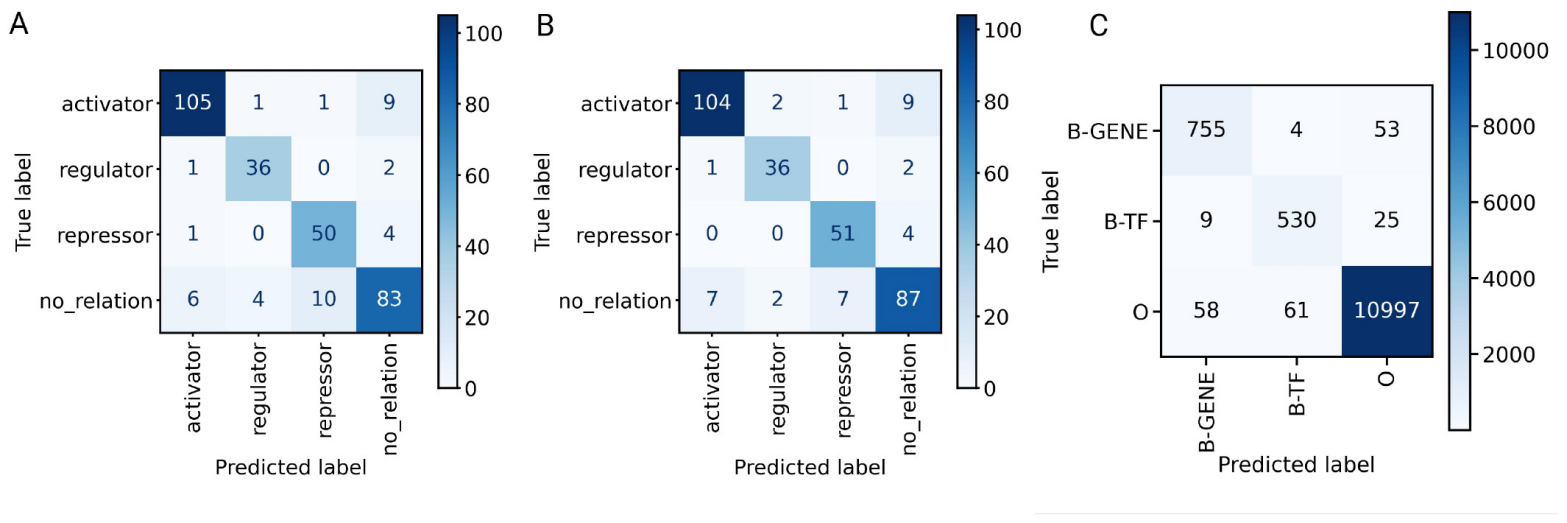
**(A)** Confusion matrix of GPT-4o mini predictions with test data set. **(B)** Confusion matrix of LLaMA-3 8B predictions with test data set. **(C)** Confusion matrix of BERN2-based algorithm predictions with test data set.

The analysis of confusion matrices of the models gives some difference in their behavior. The “repressor” category was predicted better by LLaMA-3 (8 errors) than by GPT-4o mini (11 errors) (Figure 2). Although both models misclassified the “no_relation” category more than other categories, LLaMA-3 managed to have more correct classifications. All experiments of GPT-4o mini fine-tuning and evaluation were conducted with the OpenAI API using the OpenAI service (employed hardware was unknown), the best model took 1:05:00 h. for fine-tuning. All experiments of LLaMA-3 were conducted on Google Colab Pro instances equipped with a single NVIDIA T4 (16 GB VRAM), the best model took 06:43:23 h. for fine-tuning.

### 3.2 BERN2-based NER performance

Our algorithm developed based on the BERN2 tool to recognize TFs and genes/operons achieved a high classification performance with the test data set (F1-score Macro: 0.943), with a slight tendency to a better Recall (0.953) than Precision (0.934) (Table 6); however, it failed to match the performance of the dictionary-based approach. The MCC score shows a significant performance despite the abundance of the *O* tag (MCC: 0.918). The algorithm was better at recognizing genes/operons than TFs, as the Precision score of B-GENE (0.918) surpassed the score of B-TF (0.891). By analyzing the confusion matrix (Figure 2C), we found that BERN2 predicts a considerable number of non-entity tokens as genes/operons (58 instances) and TFs (61 instances); note that some of these instances may be true entities recognized by BERN2 but absent in the entity dictionaries. We also noticed that the BERN2-based algorithm predicts more genes/operons as non-entities (53 instances) than TFs (25 instances); this results may be due to BERN2 unsuccessfully recognized the entities or the algorithm was unsuccessful to keep the BERN2 tags. Finally, we observed that the algorithm performed convincingly to distinguish TFs from genes/operons as only a few instances were misclassified: 4 genes/operons classified as TFs and 9 TFs classified as genes/operons.

**Table 6:** Metrics obtained for TFs and genes/operons NER using the BERN2-based algorithm.

Tag (entity)	Precision	Recall	F1-score	Tokens
B-GENE (genes/operons)	0.918	0.930	0.924	812
B-TF (TFs)	0.891	0.940	0.915	564
O (non-entity tokens)	0.993	0.989	0.991	11,116
Macro avg.	0.934	0.953	0.943	12,492

Despite the high performance of the BERN2-based algorithm to recognize TFs and genes/operons, we decided to utilize a dictionary-based approach for some reasons. The BERN2-based algorithm was not able to recognize all entities of the dictionaries, so we will lose TFs and regulated entities for the TRN reconstruction. This BERN2-based approach also confused TFs with genes/operons, and predicted 119 non-entity tokens as entities. Moreover, for the specific case of *Salmonella*, we have dictionaries with a considerable number of entities: 13,914 regulated elements and 471 TFs. Therefore, we preferred to apply the dictionary-based approach for *Salmonella* TRN reconstruction, leaving the application of the BERN2-based algorithm for bacteria with limited dictionaries.

### 3.3 Performance of TRN reconstruction

To evaluate the reconstruction of a TRN from 264 full-text articles of *Salmonella*, we performed the LLaMA-3 8B and GPT-4o mini best models inference with the 14349 sentences with anonymized entities. From the predictions, we discarded sentences classified in the “no_relation” category obtaining 1621 interactions for LLaMA-3 and 1510 for GPT-4o mini. To reconstruct the TRN, we obtained unique regulatory interactions (TF-regulated element-effect) from classified sentences.

**Figure 3:**
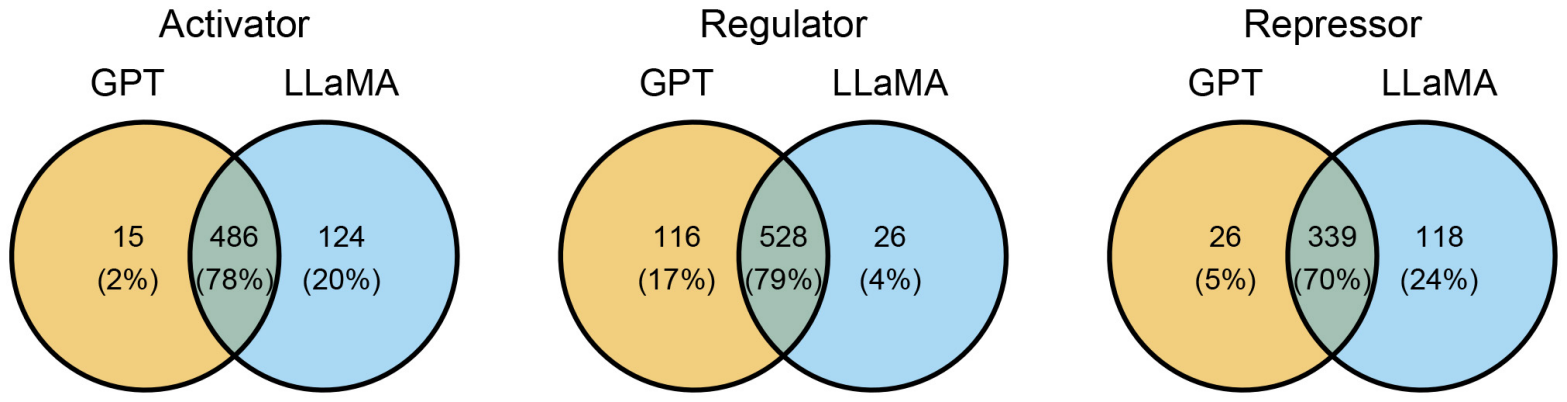
Analysis of agreement of both fine-tuned models by category in *Salmonella* TRN reconstruction from 264 articles. Intersections describe interactions extracted by both models (consensus).

Regarding the ensemble model approach, the agreement of both models reached at least 70% in the three categories (Figure 3) with a higher agreement on “activator” and “regulator” than on “repressor.” The disagreement of effect may be due to various aspects, for example, the complexity of expressing the interaction, such as in the interaction between YdgT and *ssrAB* (see following examples). The lack of a canonical regulatory verb, such as *activate, enhance, inhibit, repress*, etc., may also provoke a disagreement, see the interaction between SirB and *sirC* expressed with the phrase *the effects of* . Another cause was the lack of interaction, for example, the case of SpvR and *spvA*.

- *These involve positive regulators of* ***ssrAB*** *or the SPI2 genes, such as Fis, and negative regulators, such as H-NS and* ***YdgT***. (GPT: “repressor,” LLaMA: “activator,” true: “repressor”).
- *The effects of PhoPQ and SirB on sirC expression were also studied*. (GPT: “regulator,” LLaMA: “repressor,” true: “regulator”).
- *The lack of typical -35 elements in the* ***spvA*** *promoter regions might explain the requirement for the* ***SpvR*** *protein*. (GPT: “activator,” LLaMA: “repressor,” true: “no_relation”).

The ensemble approach outperformed individual models and previous work by obtaining an F1-Score of 0.645 (Table 7). We observed that the main drawback of these models for TRN extraction was the number of false positives reflected in the low Precision score (an in-depth analysis of false positive instances is presented in Section 4.4). Nevertheless, a great benefit of these GPT-based foundation models is that they were able to extract 80% of the complete TRN, reflected in the Recall metric. Note that the Recall score is the fraction of correctly predicted interactions of the total true interactions. This finding may be an important boost for reconstruction of new TRNs of different bacteria.

**Table 7:** Metrics obtained for *Salmonella* TRN extraction by both LLMs, ensemble model approach and previous work.

Model	TP	FP	FN	F1-score	Precision	Recall
Ensemble model approach (GPT+LLaMA)	730	623	179	0.645	0.539	0.803
GPT-4o mini	756	754	153	0.625	0.500	0.831
LLaMA 8B - Instruct bnb 4-bit	748	873	161	0.591	0.461	0.822
BERT-based model (Varela-Vega et al., 2024)	747	1,079	162	0.546	0.409	0.821

Regarding McNemar's test, Supplementary Table S2 summarizes the paired outcomes and discordant counts. comparisons between individual foundation models and the BERT-based model were not statistically significant (LLaMA vs. BERT: *p*_exact_ = 0.56; GPT vs. BERT: *p*_exact_ = 0.50), indicating that there is no evidence that models predicted true interactions at a different rate than BERT when evaluated on the same curated data set. In contrast, the ensemble model approach (agreement of LLaMA and GPT) showed a statistically significant difference with respect to BERT (*p*_exact_ = 0.016). Importantly, the direction of the discordance (*c*>*b*) indicates that BERT recovered more true interactions than the ensemble approach. This is consistent with the ensemble approach being a conservative consensus strategy. By requiring agreement, it intentionally reduces the set of predicted interactions, which can decrease false positives (increasing precision), but also increases false negatives (reducing recall) (Table 7). Therefore, statistical significance here should be interpreted as evidence that the ensemble model approach changes the recall behavior relative to BERT rather than as an overall degradation of performance. Note that LLaMA-3 and GPT-4o mini did not differ significantly (*p*_exact_ = 1.0), suggesting a similar ability to recover true/curated interactions in this evaluation.

### 3.4 Application of the approach

**Figure 4:**
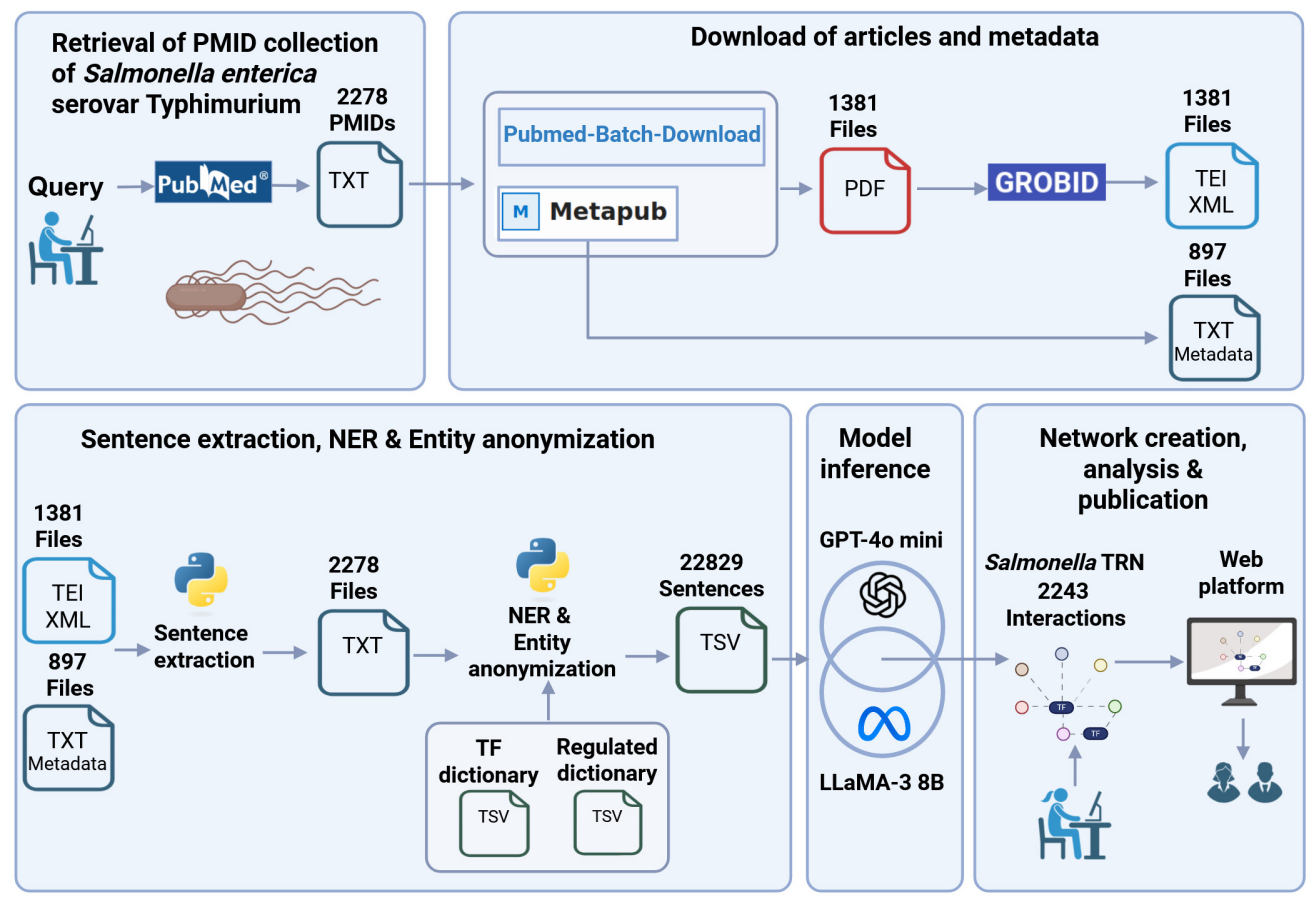
Pipeline for Salmonella TRN reconstruction using 2278 publications. Created in BioRender. Méndez, C. (2026) https://BioRender.com/p4o07c6.

We applied our approach to reconstruct a large *Salmonella* TRN using 2,278 publications, the literature available at the time (October 14, 2025) on transcriptional regulation of this bacterium. The reconstruction steps are depicted in Figure 4 and described in the following subsections.

#### 3.4.1 Retrieval of PMID collection

The selection of the PMID collection was done using the following query:

*(((salmonella enterica[Title/Abstract]) AND (typhimurium[Title/Abstract])) OR (s. enterica[Title/Abstract]) AND (typhimurium[Title/Abstract]) OR (S. typhimurium[Title/Abstract])) AND ((transcription[Title/Abstract]) OR (transcriptional[Title/Abstract]) OR (regulation[Title/Abstract]) OR (regulatory[Title/Abstract]) OR (gene expression[Title/Abstract]))*

To define the query, we considered two parts: (i) different names of the organism and (ii) keywords associated with transcriptional regulation. To determine the first part, we performed an analysis of collocations using titles and abstracts of 7,599 documents retrieved from PubMed with the query *(salmonella enterica[Title/Abstract]) AND (typhimurium[Title/Abstract])*. The collocations were defined as the most frequently co-occurring words. We reviewed collocations of two (bigrams), three (trigrams), and four (tetragrams) words (Supplementary Table S3). With this analysis, we found some variants of the name for *Salmonella enterica* serovar Typhimurium that help us to determine the keywords for the first part of the PubMed query. To determine the second part, we considered some words related to transcriptional regulation of gene expression and confirmed that these words have been used in previous related work (Gill et al., 2024). The query retrieved 2440 PMIDs on October 14, 2025, from which we removed the PMIDs of overlapping articles used to evaluate the TRN reconstruction (see Section 2.1.2). Of the 264 articles, 162 overlap with the 2,440 articles; therefore, the final PMID collection consisted of 2,278 publications.

#### 3.4.2 Download of articles and metadata

Two fetch systems were utilized to download PDF files from the PMID collection: MetaPub (https://metapub.org/) and Pubmed-Batch-Download (https://github.com/billgreenwald/Pubmed-Batch-Download). Combining the downloaded files from both systems, we gathered 1,381 full-text articles in PDF format. For the 897 publications without a PDF file, we downloaded the title and abstract (metadata) with MetaPub. The 1,381 PDF files were converted with GROBID to text format. GROBID is a machine learning tool for converting scholar documents in PDF format to structured XML/TEI documents (https://github.com/kermitt2/grobid).

#### 3.4.3 Sentence extraction, NER and entity anonymization

The 1381 XML/TEI files and the 897 metadata files comprised our collection of publications to reconstruct the Salmonella TRN (61% full-text and 39% only abstract+title). We coded a Python script to extract sentences from the XML/TEI and metadata files. As we were able to find dictionaries of TFs and regulated elements of *Salmonella*, we performed a dictionary-based NER. Then, we filter sentences with at least one TF and one regulated element. Each sentence was duplicated for each pair of TF-regulated element to obtain the data set for inference with 22,829 sentences in which the entities were replaced by the @TF$ and @Regulated$ tags (sentence with anonymized entities).

#### 3.4.4 Ensemble model approach inference

**Figure 5:**
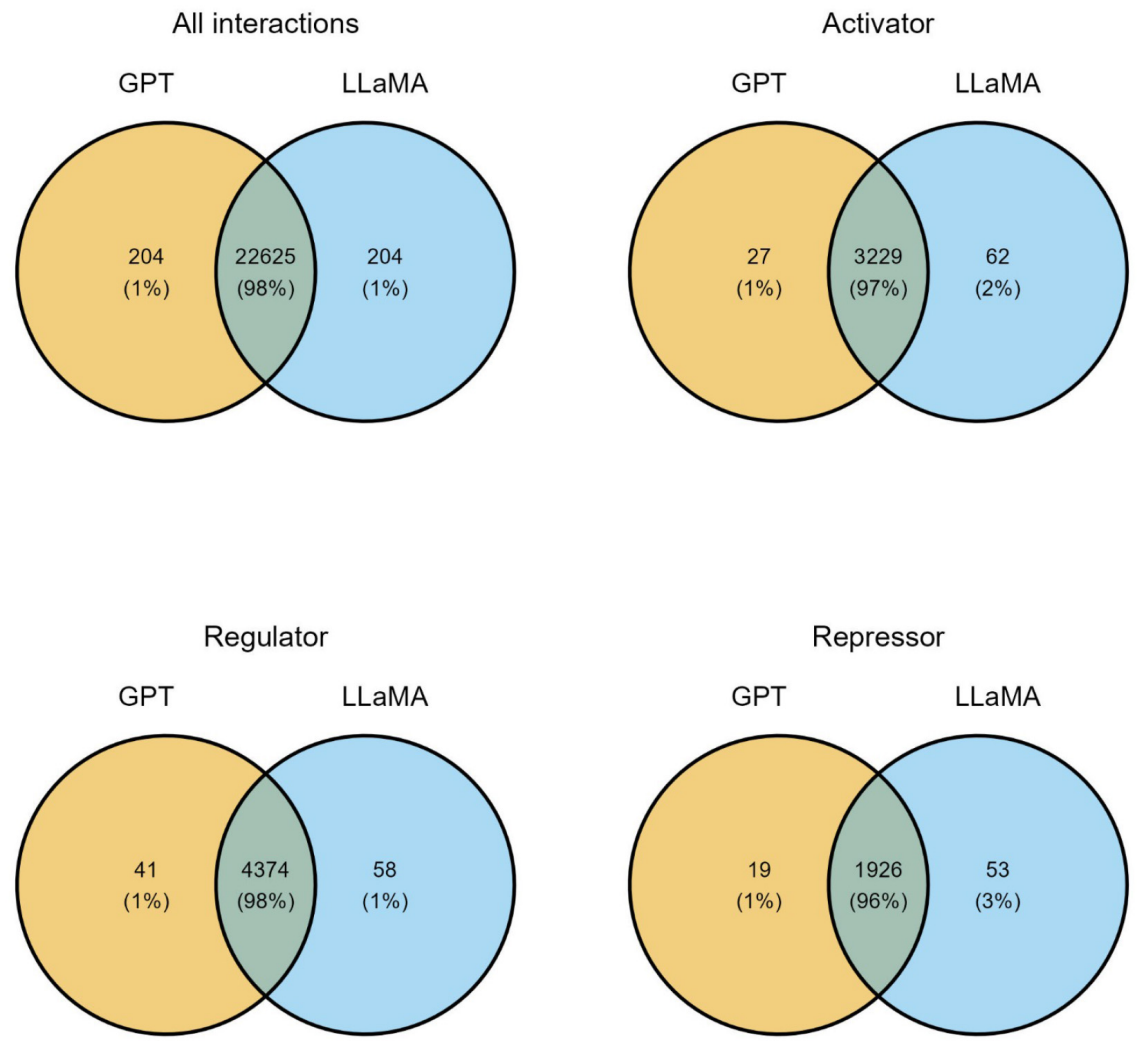
Analysis of agreement of both fine-tuned models by category in *Salmonella* TRN reconstruction from 2278 articles. Intersections describe interactions extracted by both models (consensus).

Following the ensemble model approach (Section 2.4), both fine-tuned models individually predicted the regulatory effect of the 22,829 sentences with anonymized entities. Sentences predicted with “no_relation” category were discarded. Then, unique interactions (TF-regulated element-effect) for each model were extracted from sentences. Finally, the consensus of unique interactions (models agreement) was obtained as the final set of extracted interactions by the ensemble model approach. In this case, the model agreement was very high (Figure 5). This finding becomes significant as one may opt for a close model (GPT-4o mini), or for an open model (LLaMA-3) obtaining a stable inference.

#### 3.4.5 Network creation, analyses and publication

The network creation was basically a two-step process. The first step consisted of removing interactions of “regulator” effect if the “activator” or “repressor” effect was also predicted for the same TF-regulated element. The idea behind this is that the “repressor” and “activator” effects entail the “regulator” effect. In other words, because the “regulator” category is predicted when the regulatory effect is unclear or unspecified, if the specific effect is predicted for the same interaction, the “regulator” effect may be considered redundant for the graph creation. We consider that this step may benefit downstream network analysis and visualization, because the number of edges is reduced. Note that this step is performed only for network (graph) creation, and no sentence is removed from the final set of prediction so we believe that this removal step is not a loss of information. A final set of 2,243 regulatory interactions was used for network creation. The second step was the generation of a *Salmonella* TRN graph using the Cytoscape system (Shannon et al., 2003), an open source tool system to visualize and analyze complex networks. The graph showed 1,061 nodes (TFs and regulated elements) and 2,243 edges (interactions).

Some analyses were performed to confirm that the reconstructed TRN shows biological patterns that align with existing biological knowledge, highlighting the consistency of our approach. Cytoscape was utilized to perform a network analysis to obtain the degree of nodes. The degree is the number of edges connected to a node. In a TRN, nodes with the highest degree are often TFs that regulate many elements (usually called global regulators). The connected regulated elements (community) of the TFs with the highest degree were used as separated networks for visualization with Cytoscape and over-representation analysis with the PANTHER system (Thomas et al., 2022). The over-representation analysis is one of the main analyses to obtain biological insights related to protein function. This analysis finds the most statistically significant biological processes, molecular functions, and cellular components associated with a set of genes based on Fisher's exact test. Finally, the reconstructed TRN was uploaded to an open-access Web platform for users to retrieve (https://github.com/laigen-unam/GPT-trn-reconstruction.git).

## 4 Discussion

### 4.1 Novelty of the study

Automatic extraction of transcriptional regulatory interactions of bacteria (mainly TF-gene) from the literature has previously been addressed with classical NLP and machine learning approaches. For example, some pioneer studies proposed sets of text and linguistic rules manual created to extract gene-protein interactions (Sarić et al., 2005; Rodŕıguez-Penagos et al., 2007). A similar approach based on manually defined text patterns and a formal grammar was proposed by Leitner et al. (2013). Another approach was proposed using manually defined syntactic rules to annotate the mode of interaction (activation or repressor) of sentences already classified with TF-gene interactions using ChIP-Seq (Farahmand et al., 2020).

Pattern learning approaches have also been proposed. For example, Wang et al. (2011) employed an approach to automatically learn patterns associated with manually tagged sentences categorized as positive or negative interaction, but limited only to hypoxia inducible transcription factor-1 and target genes. In the same way, Tang et al. (2011) presented a system for extracting sentences with TF-target gene interactions based on unsupervised learning of text and linguistic patterns from untagged sentences using manually defined seed patterns. Linear-chain conditional random fields to annotate regulatory interactions as a NER task was also presented in previous work (Žitnik et al., 2015). The extraction and classification of sentences with transcription regulatory interactions have also been proposed (Vazquez et al., 2022).

Despite the high precision of the rule-based approaches, a drawback is that manual creation of rules/patterns is labor intensive and difficult to update. Regarding classical Machine learning approaches, they required large training data, which is demanding to obtain. In addition, unfortunately, most of the data and implementations of previous approaches are not available. New possibilities have emerged to address NLP tasks due to recent AI-based approaches, particularly those based on foundation models, which have been a turning point in recent advancement in science and technology (Bommasani et al., 2021). Foundation models are pre-trained with a great amount of data that can be fine-tuned to solve a downstream task with limited data. This characteristic of transfer learning has presented an opportunity for NER and RE using biomedical literature (Srivastava et al., 2021).

Nevertheless, there are still few studies that have addressed the extraction of bacterial TF-gene/operon interactions with foundation models. For example, Gill et al. (2024) have recently proposed an approach to extract gene-protein interactions from filtered abstracts using two models based on the *Bidirectional Encoder Representation from Transformers* (BERT) (Devlin et al., 2019): BioBERT (Lee et al., 2019) and BERN2 (Sung et al., 2022). This approach fine-tuned a BioBERT model to filter sentences mentioning regulatory interactions from abstracts. Then, another BioBERT model was fine-tuned to classify pairs of mentions of genes-proteins as a true or false relation (binary classification). Finally, a confidence score based on the existence of the entities and the interaction in biological databases is utilized to propose the final set of interactions.

This approach for binary classification achieved outstanding performance in standard datasets. When the approach is evaluated with manual curation of a real-world database (RegulonDB) using sentences from abstracts, the performance declines. The authors reported that their method extracted 2,866 interactions, of which only 456 were correctly identified (TP = 456) in the 578 manually curated interactions (FP = 2,410, FN = 122). This involves a Precision score of 0.16, Recall of 0.79, and F1-score of 0.26. This result highlights the difficulty level to obtain high performance using real data sets. To explore the performance of our ensemble approach in the same direction, we calculated scores using a binary classification of the regulatory interactions of the TRN reconstruction from the 264 *Salmonella* articles (see Section 3.3). Our approach correctly identified 549 interactions (TP = 549) of the 860 extracted interactions in the 641 curated interactions (FP = 311, FN = 92), obtaining a Precision score of 0.64, Recall of 0.86, and a F1-score of 0.73. Thus, our approach obtained significant scores to predict true interactions with a real data set. Although a direct comparison with the work of Gill et al. (2024) is not possible since different data sets were evaluated, we may consider our method more advisable, since it accurately predicts whether an interaction is true, and it also predicts the regulatory effect.

A second related work presents a comparison of six fine-tuned BERT-based foundation models (BERT-base, BioBERT, BioLinkBERT, BioMegatron, BioRoBERTa, and LUKE) to extract TF-gene/operon interactions and classify them according to regulatory effect (regulation, activation, and repression) (Varela-Vega et al., 2024). Interestingly, LUKE, a task-specific model, outperformed the domain-specific models pre-trained with additional biomedical literature. The authors reported an F1-score of 0.546 for the *Salmonella* TRN reconstruction using the same data utilized in our study. As we used the same data, our results are comparable, resulting in a performance improvement of our ensemble model approach (+0.099). Moreover, our approach reduced false positive predictions by a considerable amount (1,079 → 623) (Table 7). Finally, two recent works have tested several GPTs models, but using the prompting strategy for human gene interactions, so they are hardly comparable to our approach (Park et al., 2025; Azam et al., 2024).

### 4.2 The reconstructed TRN integrates existing biological knowledge

One of the main drives that NLP and AI give to research work is the integration of pieces of knowledge separately published over thousands of articles. A TRN is a clear example of the result of this integration. The *Salmonella* TRN reconstructed by our approach with the 2,278 publications may support the study of this bacterium by revealing patterns of transcriptional regulation, which may lead to new hypotheses and feature studies. In this section, we discuss some of these patterns.

**Figure 6:**
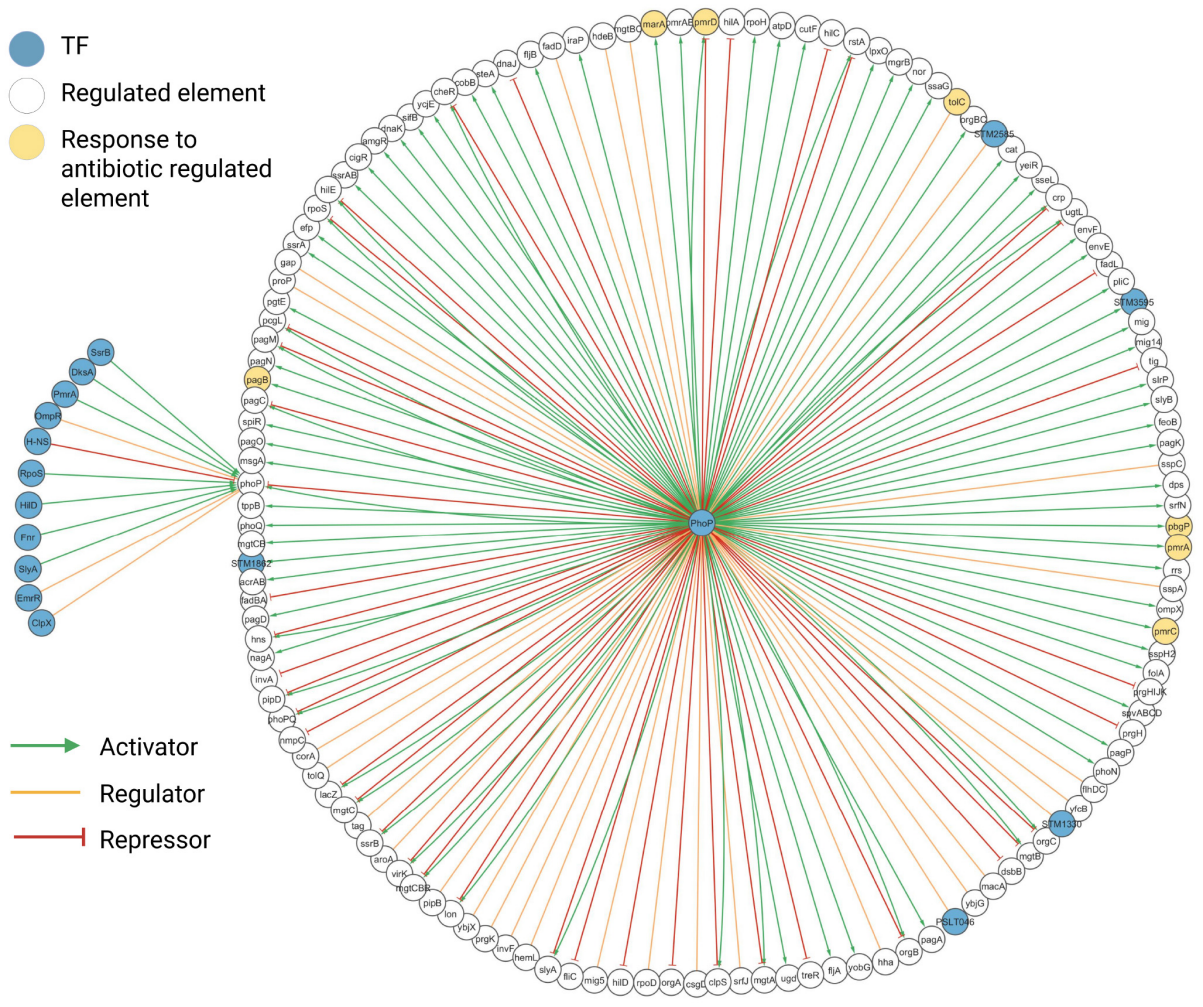
PhoP community network. Community network created with Cytoscape. Figure created in BioRender. Méndez, C. (2026) https://BioRender.com/xln5twh.

The degree value obtained with the network analysis highlighted PhoP (degree = 160), RpoS (degree = 126), and H-NS (degree = 107) as relevant TFs (Figure 6). This result aligns with existing knowledge. Phop (virulence transcriptional regulatory protein) has been described as a major regulator in *Salmonella* (Kato and Groisman, 2008), RpoS has also been described as a master regulator (Franco Meléndez et al., 2022), and H-NS has been underlined as a major repressor in this bacterium (Hurtado-Escobar et al., 2019), a fact that can be perceived in the abundance of red lines in its community. Regarding the over-representation analyses with PANTHER, the communities of the three TFs were significantly associated with several biological processes, molecular functions, and cellular components, showing that the communities comprise biological facts. Few examples are described in the following paragraphs (see complete over-representation analysis, raw Fisher's exact text *P*-values, and False discovery rate, FDR, in Supplementary Tables S4–S6).

The PhoP community over-representation analysis underlined four significant biological processes: *magnesium ion transmembrane transport, DNA-templated transcription initiation, response to antibiotic*, and *phosphorelay signal transduction system*, of which two coincided with over-represented molecular functions: *magnesium ion transmembrane transporter activity* and *phosphorelay response regulator activity*. This result aligns with existing biological knowledge, as PhoP has been reported to influence virulence together with the PhoQ sensor protein and to be activated by multiple signals or environmental variations, including low levels of Mg^2+^. PhoQ promotes the phosphorylated state of PhoP, in consequence, phosphorylation of PhoP modifies transcription in *Salmonella* (Groisman et al., 2021).

**Figure 7:**
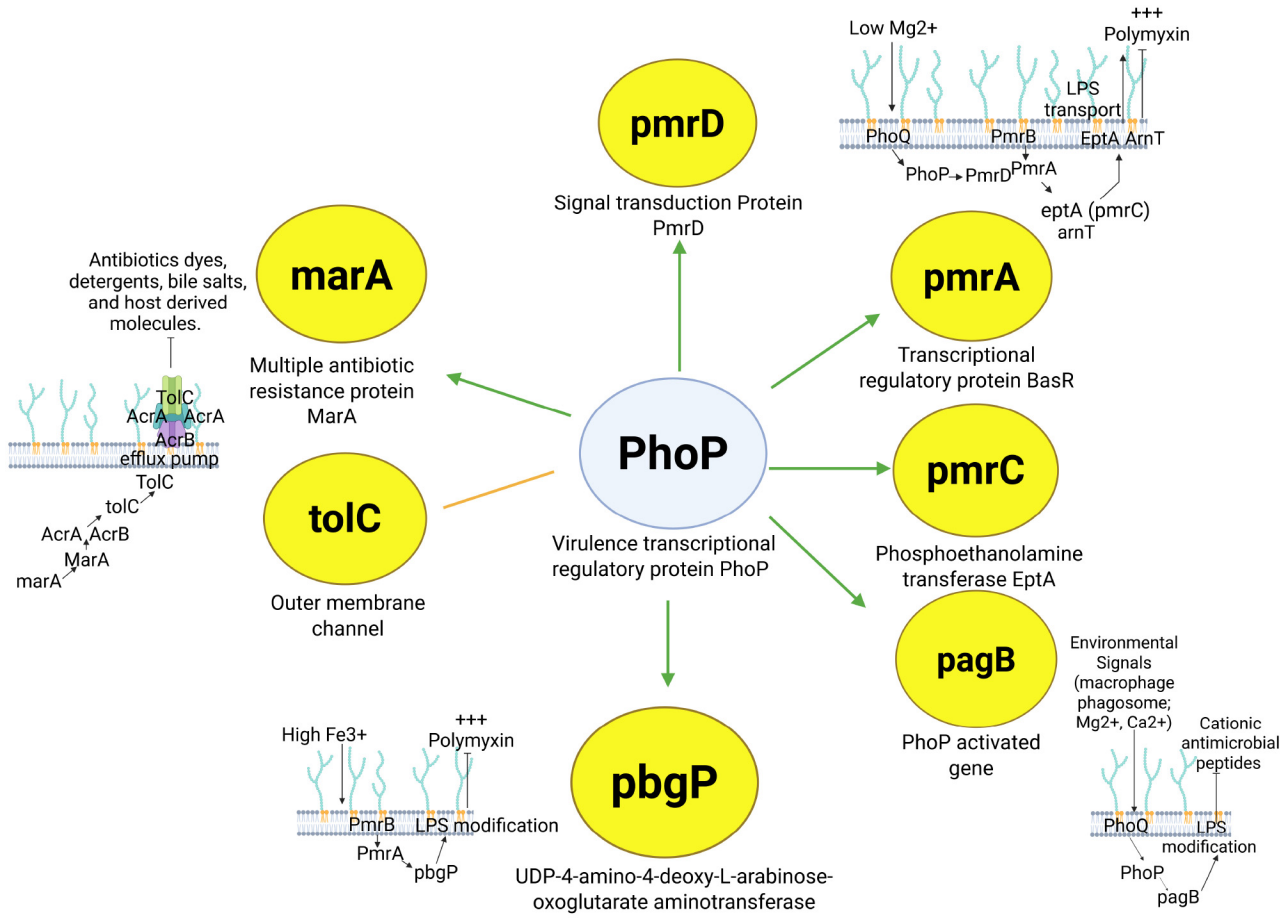
Description of PhoP-regulated response to antibiotic genes. Four transcriptional regulatory pathways related to genes are described. The green arrows represent activation, while the orange line represents regulation. Created in BioRender. Méndez, C. (2026) https://BioRender.com.

The PhoP community over-representation analysis also showed significant regulation of several genes associated with *response to antibiotic*: *marA* (multiple antibiotic resistance protein), *pagB* (Phosphoethanolamine transferase EptA), *pbgP, tolC* (outer membrane protein), and *pmrD* (Figure 6). We separated the subnetwork of these genes to explore its consistency and relevance (Figure 7). The PhoP regulatory mechanism on *pmrD, pmrA*, and *pmrC*, genes associated with antibiotic resistance, has been already described in the existing literature, a fact showing that the reconstructed network integrates existing biological knowledge. We included the reported transcriptional regulatory pathways of these genes in the same figure (see top right in Figure 7). PhoQ is activated by low concentrations of Mg^2+^, which activates PhoP and increases the transcription of *pmrD* (signal transduction Protein PmrD). The PmrD protein allows for the continuous transcription of PmrA (Transcriptional regulatory protein BasR) genes, including *eptA* (phosphoethanolamine, *pmrC*) and L-4-aminoarabinose (*arnT*). These actions include the incorporation of *eptA* and *arnT* of lipid A, which neutralize lipid A phosphates and confer resistance to polymyxin B antibiotic (Rubin et al., 2015) (Figure 7).

When *Salmonella* enters a macrophage phagosome, PhoQ senses some signals (Mag^2+^, Ca^2+^) and enhances PhoP to activate *pagB* transcription (see bottom right in Figure 7). Its transcription results in lipid A modification and cationic peptide resistance (Gunn and Miller, 1996). In the case of *pbgP* (see bottom center in Figure 7), the increase in Fe^3+^ activates PmrB and PmrA proteins, which induce the transcription of *pbgP* (UDP-4-amino-4-deoxy-L-arabinose-oxoglutarate aminotransferase), resulting in strains that lack aminoarabinose in their lipid A molecules and more susceptible to polymyxin (Lee et al., 2004).

We included in the subnetwork the description of the mechanisms of *marA* and *tolC*, although these genes are not regulated by PhoP (we confirmed that these interactions were misclassified by our model) (see left in Figure 7). The expression of *acrAB* and *tolC* (Outer membrane channel) is primarily controlled by MarA (Multiple antibiotic resistance protein). The expression of these genes encodes the AcrAB-TolC efflux pump, a system capable of ejecting antimicrobials and some metabolites (Weston et al., 2018). Despite these prediction errors, the network integrates relevant knowledge of PhoP and some regulated elements with response to antibiotic. In addition, because our approach extracted the evidence (sentences) supporting the model predictions, the cost of reviewing interactions is significantly reduced, a fact that may boost the manual curation of TRN of biological and clinical relevance.

**Figure 8:**
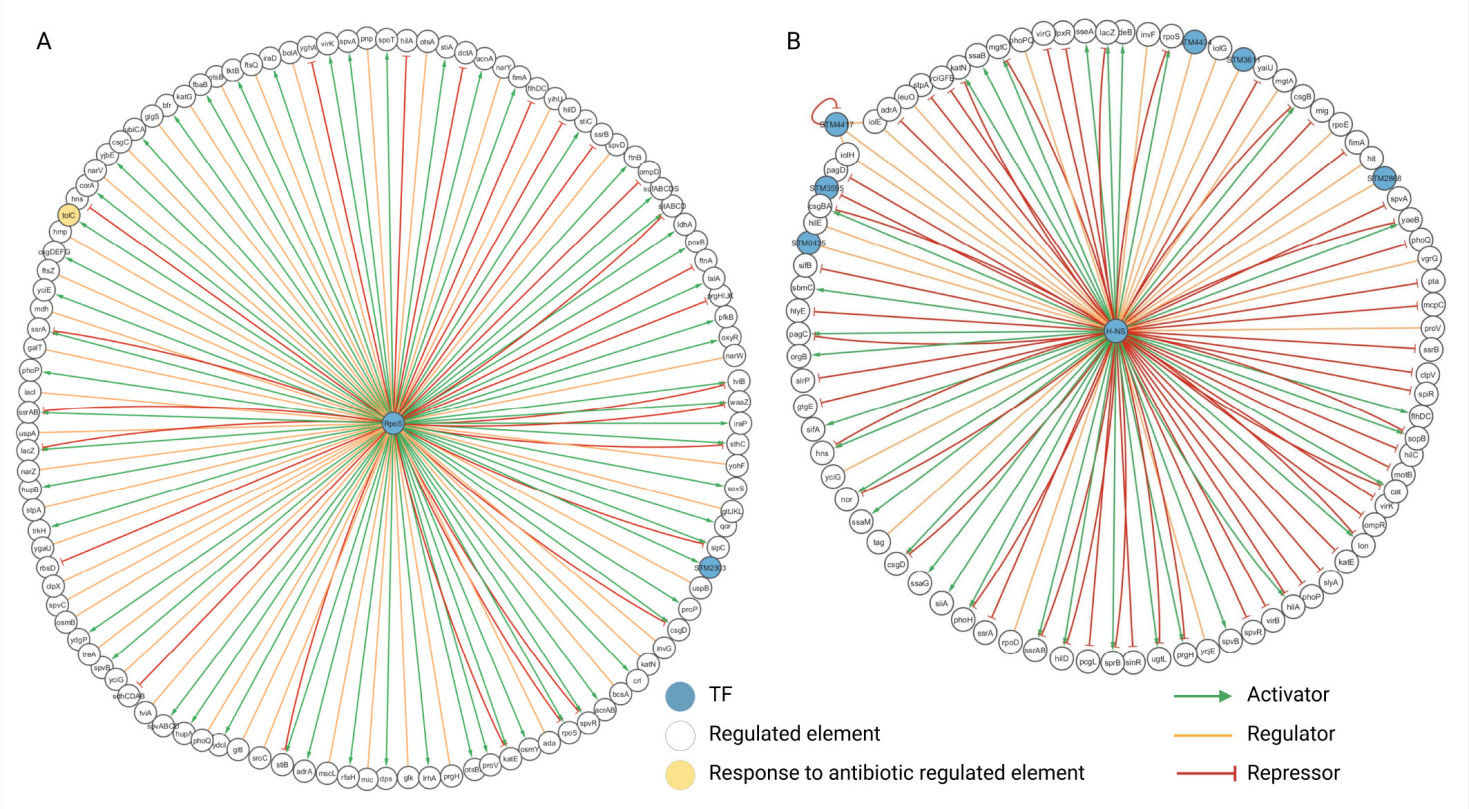
**(A)** RpoS community network. **(B)** H-NS community network. Community networks created with Cytoscape. Figure created in BioRender. Méndez, C. (2026) https://BioRender.com/jzixynb.

The over-representation analysis of RpoS proposed the biological process *cellular response to chemical stress*, which aligns with reported knowledge (Franco Meléndez et al., 2022). The Sigma factor RpoS controls the expression of genes important for systemic infection (Nickerson and Curtiss 3rd, 1997). RpoS activates pyruvate oxidase (*poxB*) and lactate dehydrogenase (*ldhA*), which are involved in important metabolic processes; these genes are presented in the reconstructed network (Figure 8A). RpoS also regulated genes involved in temperature variation, hyperosmolarity, oxidative stress, and changes in pH relevant to virulence (Franco Meléndez et al., 2022).

Lastly, the histone-like nucleoid-structuring protein (H-NS) is a nucleoid-associated protein involved in nucleoid organization and transcription regulation, also associated with virulence and adaptive stress response (Li et al., 2023). Environmental cues (temperature, pH, and metal ions) can alter the H-NS conformation to limit their activity. A known fact present in the H-NS community is that H-NS represses *phoP* (Liu and Wang, 2025) (Figure 8B).

### 4.3 Potential applications in other bacterial species

A strength of our approach is the capability to be applied to other bacterial species beyond *Salmonella*. Given the evolutionary conservation of genetic organization and regulatory mechanisms, the expression of regulatory interactions among different bacterial species should not be expected to be very different in the literature. For the reconstruction of a new TRN of a different bacterium, only TFs and regulated elements dictionaries must be required. If dictionaries were limited, applying our BERN2-based algorithm may be a way to increase them. Thanks to the fact that the model was trained with anonymized entities, changing entity names will have no impact on model predictions. Evidently, if the bacterium presented a different type of regulatory effect, the model will not be able to predict it. A middle-time objective is to apply our approach to reconstruct TRNs of diverse bacteria of clinical and biological interest.

### 4.4 Limitations and future direction of the study

A limitation of the study is the low Precision score due to the high number of false positive instances. This is a current challenge for the reconstruction of TRNs using articles, and not for standard data sets, as the problem is also present in other works (Gill et al., 2024; Varela-Vega et al., 2024). Note that in these false positives there may exist correct interactions (true positives) that may be overlooked in curation work. To understand the nature of false positives, a manual curation of 250 sentences of false positive instances was performed. These instances describe 56 unique interactions (TF-regulated element-effect). Of the 250 instances, 30% (76 instances) were correct predictions (true positives) and 70% (174 instances) were confirmed false positives. It should be noted that, among the true positives, 12 regulatory interactions were not recovered by curation work, a fact that shows the utility of our approach to support TRNs curation (Supplementary Table S7). The confirmed false positives were manually categorized into nine categories describing patterns that may explain model errors. A quantitative summary of these categories is shown in Supplementary Table S8.

*No interaction is expressed* (52%): the majority false positive instances were sentences without any regulatory interaction expressed between the TF and the regulated element. These instances should have been classified as “no_relation,” but our approach predicted different regulatory effects. We observed that several sentences describe the relation between a gene and its product (encoded TF), such as *rpoS* with RpoS.

- The **rpoS** gene encodes the alternative sigma factor S (**RpoS**) and is required for survival of bacteria under starvation and stress conditions. (Predicted category: “activator,” true category: “no_relation”).
- The **flhDC** encode the master regulator **FlhDC**. (Predicted category: “activator,” true category: “no_relation”).

*Derepression* (14%): we found several false positive instances of the regulatory effect “derepressor,” an effect not considered in our study. The model predicted the “repressor” effect for those instances, as the “derepressor” category was not considered as an output category in training data.

- *AlaC/XylS family members*, ***HilC*** *and HilD, directly bind and derepress the Salmonella typhimurium* ***hilA*** *promoter*. (Predicted category: “repressor,” true category: “derepressor”).
- *Although both* ***HilC*** *and HilD have been implicated in derepressing* ***hilA*** *expression, their effects on each other's expression are unknown*. (Predicted category: “repressor,” true category: “derepressor”).

*Lack of a regulatory verb* (11%): this category was utilized to annotate sentences expressing the regulatory interaction without a canonical regulatory verb (*activate, enhance, inhibit, repress*, etc.). We observed that most of the sentences (16/20) use the noun *effect* and the verb *affect* to express the interaction. These instances should have been classified with the “regulator” effect, but our approach inconsistently predicted the “activator” or “repressor” effect.

- *Effect of H-NS, HU, and* ***Fis*** *on* ***hilD*** *mRNA levels*. (Predicted category: “repressor,” true category: “regulator”).
- *Effect of HU and* ***Fis*** *on* ***hilA*** *expression*. (Predicted category: “activator,” true category: “regulator”).
- *The small nucleoid-binding proteins H-NS, HU, and* ***Fis*** *affect* ***hilA*** *expression in Salmonella enterica serovar Typhimurium*. (Predicted category: “repressor,” true category: “regulator”).

*Hypothetical* (10%): this category was used when the regulatory effect was hypothesized using expressions, such as *potential, we would expect*, or *we suspected*. We consider that these cases should have been classified with the “no_relation” category, because the interaction was not confirmed. We separated the cases of hedging (hedged language) from the hypothetical cases, as hedging is a strategy in academic writing to be cautious in presenting findings that could have been confirmed. Therefore, hedging cases must not be classified as “no_relation” effect; see, for example, the third sentence mentioning studies that confirmed the “regulator” effect despite the interaction being expressed by hedging.

- *Alternatively, HU or* ***Fis*** *may influence* ***hilA*** *indirectly by increasing the levels and/or activity of HilD*. (Predicted category: “activator,” true category: “no_relation”).
- *Another potential inhibitor of* ***hilA*** *expression is a protein of unknown function called* ***HilE***. (Predicted category: “repressor,” true category: “no_relation”).
- *These studies indicate that the concentrations of both* ***FimY*** *and FimZ in-vivo may be critical for* ***fimA*** *regulation*. (Predicted category: “regulator,” true category: “regulator”).

Minority categories (13%): minority categories categorized 5% of less instances. The *Complex* category (3%) was assigned when the effect is hard to infer from the sentence. For example, in the first sentence of the following examples, the recruitment of RNA polymerase indicates a “regulator” effect, but this inference depends on knowledge that the model does not have. The *Indirect regulation by another TF* category (2%) was annotated when the regulatory effect is given by the interaction with another TF (see second sentence); these cases should have been classified with “no_relation” effect. The *Prospective/To be confirmed* category (5%) was used to indicate that the interaction is proposed to be confirmed, as shown in the third sentence; these cases should also have been considered as “no_relation” effect. The minority category *Regulation by TF mutant* (1%) was used to annotate cases where the regulatory effect is expressed between a TF mutant and a regulated element; for example, in the fourth sentence, although it is clear that the TF mutant increased gene expression, the effect of the TF without mutation is not expressed. Finally, we found only one instance of the *Different organism* category (1%), which was used to categorize regulation on different bacteria, such as (*Vibrio cholerae*).

- *In the second loop*, ***FliA*** *(*σ*28) directly recruits RNA polymerase to the* ***fliA*** *promoter*. (Predicted category: “activator,” true category: “regulator”).
- *Alternatively*, ***HU*** *or Fis may influence* ***hilA*** *indirectly by increasing the levels and/or activity of HilD*. (Predicted category: “activator,” true category: “no_relation”).
- *To examine whether H-NS*, ***HU**, or Fis affects* ***hilD*** *mRNA levels, we first characterized the hilD transcript by identifying the hilD transcription start site*. (Predicted category: “repressor,” true category: “no_relation”).
- *FimW negatively regulates fimA expression, and* ***Fis*** *mutants exhibited an increased fimbriate phenotype compared to wild-type bacteria and increased* ***hilA*** *gene expression*. (Predicted category: “repressor,” true category: “no_relation”).
- ***Vibrio cholerae fur*** *mutations associated with loss of repressor activity: implications for the structural-functional relationships of* ***Fur***. (Predicted category: “repressor,” true category: “no_relation”).

The categorization of false positive instances underlines the limitations of the fine-tuning data set and the data set to evaluate TRN reconstruction. Both data sets were obtained during a real curation workflow following a strategy of *assisted curation* (Gama-Castro et al., 2014). This strategy consisted in retrieving all sentences that include at least one TF, one gene/operon, and one regulatory verb from a list of manually collected verbs (*repress, activate, inhibit, enhance*, etc.). Then, curators read the sentences and recorded only those cases where a regulatory interaction is present. These data sets have several strengths: (i) specific for bacterial transcriptional regulation, (ii) curated from full-text articles by trained curators, and (iii) It is not an artificial data set, but an example of a real data set.

The finding that many false positives were of the *No interaction is expressed* and *Lack of a regulatory verb* categories coincides with the curation strategy. Therefore, the training and evaluation data contained only true instances of interactions associated with canonical regulatory verbs (prototypical examples). We can conclude that our approach has a limited generalization to correctly predict the effect of sentences from articles that are not prototypical examples of sentences expressing regulatory interactions. A future direction is to enrich the data sets with at least the 250 categorized sentences for the model to learn patterns associated with negative, hypothetical, prospective, and non-prototypical instances. For example, if the models were re-trained with sentences mentioning encoding relations between genes and their products (TFs) classified as “no_relation” effect, the number of false positives may be reduced. In addition, we need to include the “derepressor” category during model re-training. We consider that these actions will significantly reduce the gap between test set performance (F1 = 0.892) and TRN reconstruction (F1 = 0.645), because this gap was not due to the fact that the model was trained with isolated sentences and then used to predict the effect of sentences from full-text articles, the gap is due to the fine-tuned models deals with sentences from full-text articles with expression ways that were not present in training data. The continuous in-depth analysis of sentences related to false positive predictions remains crucial to understand the limitations of foundation models to deal with the knowledge extraction from the biomedical literature.

As increasing training data with curated instances conveys considerable human effort, an alternative future direction of the study may be the integration of Natural Language Inference approaches to detect interactions with a high level of certainty, such as those used to detect assertions in medical text (Du et al., 2022). Another future direction of this study is to delve deeper into the explainability of networks. One interesting approach is the use of Graph neural networks explainability. For example, Cetin and Sefer (2025) proposed a network explainer (OExplainer) for a more comprehensively and intelligibly graphs interpretation, which was applied to protein-protein interaction datasets and outperformed related explanation approaches.

Thanks to new genomic data analysis technologies, new regulatory interactions are being published at an accelerated rate for various organisms. Given this rapid dissemination of relevant pieces of knowledge, approaches such as the one we have proposed in this work are crucial for integrating and connecting these pieces to benefit future studies on bacteria of clinical and biological interest.

## Data Availability

Original datasets are available in a publicly accessible repository: https://github.com/laigen-unam/GPT-trn-reconstruction.
