# Supplementary material for: Fine-tuned GPT-based foundation models effectively reconstruct bacterial transcriptional regulatory networks from literature
Source: Front Artif Intell. 2026 Apr 13;9:1788196. doi: 10.3389/frai.2026.1788196 (PMC13111565; doi:10.3389/frai.2026.1788196)
Supplement: Supplementary file 1 [file Data_Sheet_1.pdf]

frontiers

Supplementary Material

1 SUPPLEMENTARY METHODS

1.1 NER using BERN2

The algorithm consists of four stages described below: BERN2-based NER, tagging sentences, improving entity mentions, and rule-based classification.

1. **BERN2-based NER**
   Input sentences are automatically annotated using the local installation of BERN2.
2. **Tagging sentences**
   The algorithm filters annotations with obj == "gene" from BERN2 JSON responses and places tags <gene> and </gene> around the corresponding gene mentions within sentences. The tags are inserted carefully, sorting by index in reverse order to preserve character positions in the original text.
3. **Improving entity mentions**
   We apply the following procedures using a list of non-entity lexical items appearing before and after gene names, and lexical items indicating that the gene name is false. These lists were manually collected by observing gene mentions recognized by BERN2. These procedures were implemented using regular expressions applied to each sentence.
   - **Items before the entity:** if non-entity lexical items appear before the gene name, the opening tag is moved after them, e.g., <gene>*wild-type ArgP*</gene> → *wild-type* <gene>*ArgP*</gene>.
   - **Items after the entity:** if non-entity lexical items follow the gene name, the closing tag is moved before them, e.g., <gene>*ArgP - binding site*</gene> → <gene>*ArgP*</gene> *- binding site*.
   - **Complete mention removal (false gene names):** if complete mention appears in a list, the tags are removed, leaving the mention unannotated, e.g., <gene>*RNA polymerase*</gene> → *RNA polymerase*.
4. **Rule-based classification** In this phase, each entity mention is classified as a TF or a GENE by replacing the <gene> tag with <TF> or <GENE>. This process follows a hierarchical set of rules based on lists of lexical items collected by observing BERN2 responses:
   - If the content inside the tag contains terms associated with genes, e.g., *promoter*, *operon*, *DNA*, *mutant*, it is classified as <GENE>.
   - If the content inside the tag contains TF-related words, e.g., *protein*, *repressor*, *activator*, it is classified as <TF>.
   - If no rule is applied, morphological patterns are evaluated.
     - Mentions starting with lower case and ending with uppercase letters (e.g., "cyaA") are classified as genes.
     - Mentions starting with uppercase followed by lowercase and ending in uppercase (e.g., "ArcA") are classified as TFs.
   - If none of the above conditions are met, the tags are removed, leaving the mention unannotated.

   This combined strategy minimizes false positives without requiring labor-intensive manual supervision. The use of linguistic rules, together with structural patterns, provides flexibility to adapt to variations in entity naming across bacterial contexts.

5. Evaluation

2 SUPPLEMENTARY TABLES AND FIGURES

2.1 Tables

Table S1. Fine-tuning metrics obtained with 5-fold cross validation. Values are reported as mean, ±standard deviation, together with 95% confidence intervals (CI). Precision, Recall, and F1-score are macro-averaged; MCC is computed in the multiclass setting.

Model	F1-score	Precision	Recall	MCC
GPT-4o mini	0.886±0.0189	0.890±0.0232	0.886±0.0198	0.848±0.0193
$CI_{95\%}$	[0.861–0.911]	[0.861–0.919]	[0.861–0.911]	[0.824–0.872]
LLaMA-3 8B – Instruct bnb 4-bit	0.886±0.0169	0.894±0.0174	0.883±0.0198	0.848±0.0246
$CI_{95\%}$	[0.865–0.907]	[0.872–0.916]	[0.859–0.908]	[0.817–0.878]

Table S2. McNemar 2×2 contingency tables computed over unique true/curated TF-regulated element pairs for pairwise model comparisons. Each true interaction was treated as a binary outcome (predicted / not predicted), independently of the regulatory effect. Counts a, b, c, and d correspond to paired agreements and disagreements used to compute McNemar's test.

Comparison (A vs. B)	a	b	c	d
LLaMA vs. BERT	544	11	15	71
GPT vs. BERT	547	8	12	74
Ensemble (LLaMA+GPT) vs. BERT	537	8	22	74
LLaMA vs. GPT	549	6	6	80

Table S3. Top ten results of the analysis of collocations using titles and abstracts of 7599 documents retrieved from PubMed.

Bigrams
('Salmonella', 'enterica')
('enterica', 'serovar')
('serovar', 'Typhimurium')
('S', 'Typhimurium')
('S', 'enterica')
('Salmonella', 'Typhimurium')
('Escherichia', 'coli')
('E', 'coli')
('Department', 'Microbiology')
('Veterinary', 'Medicine')
Trigrams
('enterica', 'serovar', 'Typhimurium')
('Salmonella', 'enterica', 'serovar')
('S', 'enterica', 'serovar')
('Salmonella', 'enterica', 'serotype')
('serovar', 'Typhimurium', 'S')
('enterica', 'subsp', 'enterica')
('Salmonella', 'enterica', 'subsp'),
('enterica', 'serotype', 'Typhimurium')
('Typhimurium', 'S', 'Typhimurium')
('College', 'Veterinary', 'Medicine')
Tetragrams
('Salmonella', 'enterica', 'serovar', 'Typhimurium')
('S', 'enterica', 'serovar', 'Typhimurium')
('enterica', 'serovar', 'Typhimurium', 'S')
('Salmonella', 'enterica', 'subsp', 'enterica')
('Salmonella', 'enterica', 'serotype', 'Typhimurium')
('serovar', 'Typhimurium', 'S', 'Typhimurium')
('Salmonella', 'enterica', 'serovar', 'typhimurium')
('Salmonella', 'enterica', 'Serovar', 'Typhimurium')
('enterica', 'subsp', 'enterica', 'serovar')
('type', 'III', 'secretion', 'system')

Table S4. Results of the PhoP community over-representation analysis with PANTHER.

Term	Raw Fisher's Exact test *P*-value	False discovery rate (FDR)
	Biological processes	
magnesium ion transmembrane transport	$5.03e^{-05}$	$7.06\ e^{-03}$
DNA-templated transcription initiation	$4.18e^{-04}$	$3.27\ e^{-02}$
response to antibiotic	$3.45e^{-04}$	$3.13e^{-02}$
phosphorelay signal transduction system	$3.69e^{-04}$	$3.22e^{-02}$
	Molecular functions	
magnesium ion transmembrane transporter activity	$5.03e^{-05}$	$1.35e^{-02}$
sigma factor activity	$2.43e^{-04}$	$4.01e^{-02}$
DNA-binding transcription activator activity	$1.60e^{-04}$	$2.86e^{-02}$
phosphorelay response regulator activity	$3.71e^{-05}$	$1.14e^{-02}$
transcription cis-regulatory región binding	$8.12e^{-06}$	$5.82e^{-03}$
	Cellular components	
sigma factor antagonist complex	$5.52e^{-04}$	$8.14e^{-03}$
extracelular region	$2.03e^{-08}$	$1.20e^{-06}$
protein-DNA complex	$2.82e^{-05}$	$8.33e^{-04}$
cell outer membrane	$9.48e^{-05}$	$2.49e^{-03}$

Table S5. Results of the RpoS community over-representation analysis with PANTHER.

Term	Raw Fisher's Exact test *P*-value	False discovery rate (FDR)
	Biological processes	
reactive nitrogen species metabolic process	$2.44e^{-05}$	$2.77e^{-02}$
cellular response to chemical stress	$1.97e^{-05}$	$4.46e^{-02}$
regulation of cellular process	$4.4e^{-05}$	$3.38e^{-02}$
	Molecular functions	
structural constituent of chromatin	$2.02e^{-06}$	$4.34e^{-03}$
	Cellular components	
DnaA-HU complex	$3.81e^{-04}$	$1.12e^{-02}$
cytosol	$1.77e^{-03}$	$2.99e^{-02}$

Table S6. Results of the H-NS community over-representation analysis with PANTHER.

Term	Raw Fisher's Exact test *P*-value	False discovery rate (FDR)
	Biological processes	
biological regulation	$2.40e^{-10}$	$2.72e^{-07}$
DNA- templated transcription initiation	$7.07e^{-05}$	$8.01e^{-03}$
response to abiotic stimulus	$7.99e^{-04}$	$4.53e^{-02}$
phosphorelay signal transduction system	$4.05e^{-04}$	$2.55e^{-02}$
	Molecular functions	
minor Groove of adenine-thymine-rich DNA binding	$1.67e^{-04}$	$3.25e^{-02}$
sigma factor activity	$4.08e^{-05}$	$8.5e^{-03}$
molecular transducer activity	$2.8e^{-04}$	$4.74e^{-02}$
transcription cis-regulatory region binding	$2.41e^{-06}$	$1.03e^{-03}$
	Cellular components	
sigma factor antagonist complex	$1.67e^{-04}$	$3.93e^{-02}$
extracellular region	$5.12e^{-04}$	$4.03e^{-02}$
protein-DNA complex	$2.38e^{-04}$	$2.81e^{-02}$

Table S7. Regulatory interactions predicted by our ensemble approach that were not recovered by curation work.

#	Regulator	Target	Relation
1	FimW	fimA	activator
2	FimW	fimA	regulator
3	FimW	fimA	repressor
4	FimZ	fimW	regulator
5	Fis	hilA	activator
6	Fis	hilA	repressor
7	HilE	hilA	repressor
8	HU	hilA	activator
9	HU	hilA	regulator
10	HU	hilA	repressor
11	YdgT	ssrB	repressor
12	FliA	fliA	activator

Table S8. Quantitative summary of manually categorized false positives. The nine categories describe patterns that may explain model errors. Sort by number of instances.

#	Category	Instances	Percentage
1	No interaction is expressed	90	52%
2	Derepression	25	14%
3	Lack of a regulatory verb	20	11%
4	Hypothetical	17	10%
5	Prospective/To be confirmed	9	5%
6	Complex	6	3%
7	Indirect regulation by another TF	4	2%
8	Regulation by TF mutant	2	1%
9	Different organism	1	1%
Total	174	100%	
